# Integrated analyses of the methylome and transcriptome to unravel sex differences in the perirenal fat from suckling lambs

**DOI:** 10.3389/fgene.2022.1035063

**Published:** 2022-11-01

**Authors:** Pablo A. S. Fonseca, María Alonso-García, Rocio Pelayo, Hector Marina, Cristina Esteban-Blanco, Javier Mateo, Beatriz Gutiérrez-Gil, Juan-José Arranz, Aroa Suárez-Vega

**Affiliations:** ^1^ Departamento de Producción Animal, Facultad de Veterinaria, Universidad de León, León, Spain; ^2^ Departamento de Higiene y Tecnología de Los Alimentos, Facultad de Veterinaria, Universidad de León, León, Spain

**Keywords:** RNA sequencing, whole-genome bisulfite sequencing, suckling lambs, omics integration, fat deposits, perirenal fat

## Abstract

In sheep, differences were observed regarding fat accumulation and fatty acid (FA) composition between males and females, which may impact the quality and organoleptic characteristics of the meat. The integration of different omics technologies is a relevant approach for investigating biological and genetic mechanisms associated with complex traits. Here, the perirenal tissue of six male and six female Assaf suckling lambs was evaluated using RNA sequencing and whole-genome bisulfite sequencing (WGBS). A multiomic discriminant analysis using multiblock (s)PLS-DA allowed the identification of 314 genes and 627 differentially methylated regions (within these genes), which perfectly discriminate between males and females. These candidate genes overlapped with previously reported QTLs for carcass fat volume and percentage of different FAs in milk and meat from sheep. Additionally, differentially coexpressed (DcoExp) modules of genes between males (nine) and females (three) were identified that harbour 22 of these selected genes. Interestingly, these DcoExp were significantly correlated with fat percentage in different deposits (renal, pelvic, subcutaneous and intramuscular) and were associated with relevant biological processes for adipogenesis, adipocyte differentiation, fat volume and FA composition. Consequently, these genes may potentially impact adiposity and meat quality traits in a sex-specific manner, such as juiciness, tenderness and flavour.

## Introduction

Sheep meat, like the meat of other ruminants, is a good source of beneficial fatty acids (FAs) for human health, such as polyunsaturated fatty acids (PUFAs), branched-chain FAs, PUFA biohydrogenation intermediates, and conjugated linolenic acids, among others (Dilzer and Park, 2012; Ran-Ressler et al., 2014). The meat from suckling lambs is characterised by its tenderness, low-fat levels, moisture and pale pink colour, which result in a high-quality and valuable product in Mediterranean countries (Sañudo et al., 2007). Therefore, sheep meat meets the increasing demand from the consumer market for products that combine quality and health-promoting properties (de Smet and Vossen, 2016).

Fat deposits are a fundamental part of the organoleptic quality of carcasses. In general, adipose tissues are differentially regulated in males and females partly due to the effect of androgens and oestrogens, especially testosterone (Clegg et al., 2006; McInnes et al., 2006; Clarke et al., 2012). In sheep, differences were observed in the metabolic homeostasis response during changes in the growth, muscle and fat accumulation rates between males and females (Rosales Nieto et al., 2019). Additionally, sex effects on specific FA profiles (i.e., higher levels of PUFA and ꞷ3 in males) and meat tenderness (i.e., tender meat for females) were also described in sheep (Vargas Junior et al., 2019). More specifically, in the Assaf breed, females reach the phase of greater fat deposition at lower body weights, with sex differences evident at very low body weight (Landa et al., 2004).

In lambs, the FA composition of adipose deposits is directly influenced by the composition of the milk and/or supplementary feedstuffs they consume (Doney et al., 1984; Zygoyiannis et al., 1992; Bas and Morand-Fehr, 2000; Frey et al., 2001; Osorio et al., 2007). However, it has been described that in suckling lambs, independent of diet, females are predisposed to develop more fat than males (Velasco et al., 2000; Dervishi et al., 2012). Moreover, other research in elder lambs (11 weeks) showed that body fat content was higher in females than in males, and in perirenal fat, females had larger adipocytes (Landa et al., 2004). Another factor to consider during the first 2 weeks of postnatal life is that the perirenal adipose deposit transits from predominantly brown adipose tissue (BAT) in the first 4 days of life to predominantly white adipose tissue (WAT) at approximately 14 days of life (Basse et al., 2015). However, some studies from our research group confirmed the presence of brown adipocytes in suckling lambs slaughtered between 17 and 23 days of life (Suárez-Vega et al., 2018). This transition from BAT to WAT is guided by the regulation of different genes and transcription factors, which reflects the necessity of the organism to move from thermoregulation in the first days of life to growth and homeostasis in the next stages of life. Interestingly, sex-specific differences in BAT mass, distribution, and activity have been identified in humans and rodents, with females having more BAT than males (Kaikaew et al., 2021).

On the basis of the foregoing information, the investigation of the biological mechanisms associated with the differences between males and females regarding fat metabolism has the potential to improve the knowledge about genes and biological processes that drive such sex differences, consequently helping to discover potential targets to improve the production, quality, and food safety of sheep products. Until now, most studies aiming to characterise gene expression differences in adipose tissue between male and female lambs have focused on studying candidate genes using real-time PCR analysis (Velasco et al., 2000; Muhlhausler et al., 2008; Dervishi et al., 2012). However, high-throughput sequencing technologies have revolutionised the field of molecular biology by enabling large-scale sequencing to explore the intrinsic mechanisms of tissue directly at the DNA or RNA level. In this sense, the integration of different omics technologies through systems biology is postulated as a relevant approach for the investigation of biological and genetic mechanisms associated with complex traits, such as nutritional status and metabolism (Zhang et al., 2008; Karahalil, 2016; Nielsen, 2017). The informative potential of these tools is based mainly on the capacity to analyse and scrutinise different levels of biological information in an integrative way. For example, the genome and transcriptome can be analysed regarding the differences in the regulatory mechanisms using whole-genome bisulfite sequencing (WGBS) and RNA-sequencing (RNA-seq), respectively. In livestock animals, such an integrative approach allowed the identification of candidate genes and biological processes associated with several relevant traits, such as fertility (cattle), sex-specific pubertal development (goats), skeletal muscle development (sheep) and fat deposition (broilers) (Yang et al., 2016; Fan et al., 2020; Gross et al., 2020; Gong et al., 2021).

The combination of both datasets in an experimental design contrasting sexes is hypothesized to help identify candidate genes responsible for controlling meat quality traits of sheep products. This hypothesis is mainly grounded on the importance of adipose deposits and the differences observed between males and females. Therefore, the objectives of this work were 1) to evaluate sex-specific methylation marks (by WGBS) and gene expression patterns (by RNA-Seq) in the perirenal fat of suckling lambs and 2) to integrate both omics approaches to identify candidate genes and biological processes underlying sex differences in carcass fatness and meat quality in sheep.

## Materials and methods

### Ethics statement

The lambs included in this experiment were not subjected to any experiment, and their management was carried out following the usual management practices on farms raising suckling lambs with artificial lactation. The management, transport and slaughter of the animals at a local slaughterhouse were in accordance with Spanish and EU legislation [Spanish Laws 32/2007, 6/2013 and RD 37/2014; Council Regulation (EC) 199/2009].

### Samples

Perirenal fat samples were collected from 12 Assaf suckling lambs (6 males and 6 females). All animals were born at the Instituto de Ganadería de Montaña (IGM) (Grulleros, León, Castilla y León, Spain) in the same lambing season (winter 2019–2020) from primiparous Assaf dairy ewes. The lambs were reared under the same management and diet conditions. Initially, during the first 24 h after lambing, the lambs were kept with their dams to suck the colostrum. Subsequently, the animals were held in lamb pens fed with artificial milk using Ovilac 60 (Calfvet^®^) milk replacer powder *ad libitum* up to slaughter to remove the influence of maternal effects. All the animals are progeny from different ewes; however, only two rams are among the parents of the animals composing the current sample. Five females and four males were paternal half-sibs, while one female and two males shared a second ram. The lambs were slaughtered at a local slaughterhouse at the market weight for the PGI label “lechazo de Castilla y Leon” (9–12 kg), which has average ages of 28.16 (±4.87) and 24.16 (±5.19) for males and females, respectively. All the animals had the percentage of perirenal, subcutaneous and intramuscular fat measured as described by (Mateo et al., 2018).

The mean percentages of renal, pelvic, renal and pelvic, leg intramuscular, and leg subcutaneous fat were compared between males and females using a Student’s t test. For these analyses, variance equality was assumed after an F test, and normality was assumed after an Anderson‒Darling test. The abovementioned statistical analyses were performed using R software (R Core Team, 2021), and the significance threshold was defined as a *p*-value <0.05 for all analyses.

### RNA extraction and RNA-sequencing analysis

For RNA preservation, the sampled tissues were preserved in an RNA-stabilization solution (Ambion RNAlater; Life Technologies) and stored at 4°C for 24 h. Subsequently, the RNA-stabilization solution was removed, and the samples were frozen at -80°C until RNA extraction. RNA was extracted using the miRNeasy Mini KIT (Qiagen, Germany) with adaptations for use in adipose tissue (up to 100 mg of tissue and inclusion of Qiagen RNeasy Lipid Tissue Mini Kit). The Agilent 2,100 Bioanalyzer device (Agilent Technologies, CA, USA) was used to estimate the RNA integrity value, which was higher than 7 for all the samples. The UltraTM RNA Library Prep Kit (NEBNext^®^, MA, USA) was used for cDNA library construction at Novogene in Cambridge (UK). The 12 cDNA libraries were sequenced on an Illumina Novaseq 6,000. A minimum depth of 30 million 150 bp stranded paired-end reads was generated for each sample. The raw datasets derived from the sequencing are available at ArrayExpress repository with reference E-MTAB-12130.

### RNA-sequencing quality control, mapping and quantification

The quality control of RNA-Seq reads was performed using FastQC version 0.11.8 (Andrews, 2015) to identify potential sequencing artefacts, duplicated sequences, adapters and base quality (Phred score) distribution. Next, the raw reads were processed and trimmed by Trimmomatic (version 0.38) to remove Illumina adapters, low-quality bases (Phred <30), reads with an average quality score below 30 within a sliding window of four nucleotides and reads with less than 75 bp after trimming. After quality control, the remaining reads were aligned to the ovine reference genome Oar_Ram_v2.0 (annotation release 104) available at the National Center of Biotechnology Information (NCBI) (https://www.ncbi.nlm.nih.gov/assembly/GCF_016772045.1/) using the software STAR version 2.7.10a (Dobin et al., 2013) with default parameters. Transcript quantification was performed using the software RSEM version 1.1.17 (Li and Dewey, 2011).

### Differentially expressed gene analysis and weighted gene correlation network analysis

The read counts were normalised using Fragments Per Kilobase per Million Mapped Reads (FPKM). Gene transcripts with FPKM<0.2 in both males and females were removed from the analysis. After filtering low expressed genes, the raw read counts for the remaining genes were normalised and fitted in a model contrasting females and males using a negative binomial distribution in DESeq2 (Love et al., 2014) with females as reference group for the contrasting. The raw reads counts were normalized using the median of radios method implemented in DESeq2 for differential expression analysis. Once the differential expression analysis was performed, the *p* values were adjusted by multiple testing using the Benjamini and Hochberg False-Discovery Rate (FDR) method (Benjamini and Hochberg, 1995). The differentially expressed genes (DEGs) were identified using a threshold composed of FDR<0.05 and |log2(Fold-Change)| >2.

Independent of the differential gene expression analysis, the FPKM normalised read counts were used to identify correlated gene networks using the R package CoExpNets (https://github.com/juanbot/CoExpNets). The CoExpNets package is based on the WGCNA R package (Langfelder and Horvath, 2008); however, it applies an additional step where the genes are reallocated within modules using a k-means clustering approach, which results in modules with a higher biological meaning (modules composed by genes which play more similar functional roles when compared with the traditional module assignment provided by the WGCNA package) (Botía et al., 2017). Previously, to construct the correlated gene networks, the WGCNA R package was used to identify genes and/or samples with too many missing entries and genes with zero variance between the male and female datasets. For this purpose, the function goodSamplesGenesMS() was used with the default options. The coexpressed gene networks were identified by the getDownstreamNetwork () function from the CoExpNets package using 20 iterations and signed networks. The coexpressed modules detected for each group were compared using the following methodology:

- For each sample (s) in male and female;
- For each module m(s) in s;
- Apply a Fisher’s exact test under the null hypothesis that there is no significant overlapping of m (male) in females and m (female) in males after an FDR 5% adjustment.

The modules of coexpressed genes in males without a counterpart in females and *vice versa*, here called DcoExp modules, were selected.

### DNA extraction and whole-genome bisulfite sequencing analysis

The DNA samples were extracted from the perirenal fat using the QuickGene DNA Tissue Kit (Autogen, MA, USA) based on protein removal by protease K following the manufacturer’s instructions. The DNA samples had an A260/280 ratio >1.8, indicating a high quality for sequencing. The samples were used for paired-end (150 bp) library construction on Novogene in Cambridge (UK). Library construction was performed using the EZ DNA Methylation Gold Kit (Zymo Research). Initially, the genomic DNA was fragmented into 350 bp fragments through sonification. Subsequently, the methylated cytosines were converted to thymine by bisulfite conversion. After this step, the Accel-NGS Methyl-Seq DNA Library Kit (Swift Biosciences) was used to prepare the libraries for WGBS, generating postbisulfite libraries. The libraries were sequenced on an Illumina NovaSeq 6,000, with a minimum coverage depth of 20X for each sample. The raw datasets derived from the sequencing are available at European Nucleotide Archive (ENA) repository with accession number PRJEB55533.

### Methylation calling and differentially methylated region identification

The quality control of reads generated by the WGBS was performed using FastQC (Andrews, 2015). Trim Galore software (version 0.6.5) (Krueger, 2015) was used to trim the reads based on quality scores, remove adapters and filter short reads using the default options. The ovine reference genome Oar_Ram_v2.0 was indexed using BowTie2 (Langmead and Salzberg, 2012). Subsequently, the trimmed reads were aligned to the reference genome using Bsseeker2 (Guo et al., 2013) software by the Python script bs_seeker2-align.py using the default options. The alignment output files were sorted by position using Samtools software (version 1.15.1) (Li et al., 2009). After this step, the duplicated reads were removed using Picard software (version 2.25) (https://broadinstitute.github.io/picard/). The Python script bs_seeker2-call_methylation.py from Bsseeker2 was used to perform the methylation calling procedure using the default options.

The R package DSS was used to identify differentially methylated loci (DMLs) and differentially methylated regions (DMRs) (Feng et al., 2014). A DML corresponds to a differential methylation pattern in a single nucleotide, while a DMR represents a differential methylation pattern in a group of nucleotides. Initially, the mean methylation levels for all the methylated sites were estimated using a simple average algorithm for smoothing, as described by (Feng et al., 2014). Subsequently, the dispersion at each methylated site was estimated, and a Wald test was conducted to identify the DMLs with a *p* value threshold of 0.001. The DMRs were detected based on regions with many statistically significant methylated sites based on the following criteria: *p* value <0.01 for the methylated site, minimum length (50 bp), minimum number of methylated sites (3), and percentage of methylated sites being significant in the region (0.5). The DMRs mapped in regions less than 50 bp from each other were merged into a single DMR. The identified DMRs were annotated using the R package genomation (Akalin et al., 2015) using the gene annotation from the ovine reference genome Oar_Ram_v2.0 (annotation release 104). First, the DMRs were mapped on promoter, intron and exon regions of the respective genes. Additionally, when the DMR was not mapped within a gene coordinate, the closest gene was assigned to the DMR, and the same was classified as mapped in an intergenic region. In general, the methylation can occur in three different contexts in eukaryotes: CG, CHG, CHH (where H is C, T or A) (Bird, 2002; Cokus et al., 2008; Lister et al., 2009). The CG context is the most observed with a range of 60%–90% of all CG dinucleotides methylated in the genome (Ehrlich et al., 1982; Bird, 1986; Lister et al., 2008). In addition, most of the CG-rich regions, also called CpG islands acting in gene silencing activities due to the overlap with proximal promoters (Suzuki and Bird, 2008). However, the importance of methylated sites outside promoter regions for the regulation of gene silencing and activation is not neglectable due to the activity of regulatory elements such as silencers and enhancers (Wu et al., 2010; Lou et al., 2014; Maegawa et al., 2017; Smith et al., 2017; Brandão et al., 2018; Ordoñez et al., 2019). The sex effect for global methylation in the CG, CHG and CHH contexts was estimated by Cohen’s D using the R package lsr.

### Integrating multiomics using multiblock (s)PLS-DA to predict male and female samples

A supervised learning analysis was conducted to identify gene expression profiles and the methylation levels for the DMRs mapped within the three contexts, which better classified the samples as males or females. This approach is called Data Integration Analysis for Biomarker discovery using Latent variable approaches for Omics studies (DIABLO) or multiblock (s)PLS-DA, and it is implemented in the R package mixOmics (Rohart et al., 2017). The method applied by DIABLO is partly based on generalised canonical correlation analysis to perform an N-integration of datasets (omics or not) with sparse discriminant analysis to classify discrete outcomes. For this multiomics integration, all the genes with |log2(Fold-Change)| > 1 in the RNA-Seq data and the detected DMRs for WGBS data were used. The analysis was performed with the gene expression values and the mean methylation level of cytosines within DMRs. The two main components were used for the discriminant analysis in both datasets. The function selectVar() from the mixOmics package was used to identify the selected variables used to discriminate the samples from each dataset. To evaluate the potential of these genes to classify males and females, the area under the curve (AUC) for the discriminant analysis was estimated using the pairs of expression values and methylation levels from the abovementioned genes exclusively.

### Identification of candidate differentially coexpressed gene modules correlated with percentage of fat in different fat deposits

The DcoExp modules harbouring at least one of the genes among the selected variables in the discriminant analysis were selected. From these DcoExp modules, the module eigengenes (MES) were extracted. The WGCNA R package was used to estimate the Pearson correlation between the MES for the selected DcoExp modules and the percentages of renal, pelvic, pelvic and perirenal, leg subcutaneous, and leg intramuscular fat. Significant correlations were defined based on a *p* value threshold of <0.05. It is important to highlight that the correlations were estimated between the pairs of modules and traits within the corresponding group. In other words, only observations from males were used to estimate the correlation with exclusively male DcoExp modules and observations from females for exclusively female DcoExp modules. Finally, DcoExp modules harbouring genes selected on the DIABLO analysis and significantly correlated with at least one of the evaluated fat deposits (percentage of perirenal, subcutaneous and intramuscular fat) were selected as candidate DcoExp modules.

### QTL overlapping and gene ontology analysis

The list of candidate genes obtained from DIABLO discriminant analysis was annotated for the overlapping of quantitative trait loci (QTL) based on the SheepQTLdb from Animal QTLdb (Hu et al., 2019), and a QTL enrichment analysis was performed using the GALLO R package (Fonseca et al., 2020). Additionally, the R packages ClusterProfiler (Yu et al., 2012) and enrichplot were used for Gene Ontology (GO) term enrichment analysis, graphic representation and functional grouping of GO terms. QTL and GO terms were considered enriched when the False-Discovery Rate (FDR) was <0.05. GO term enrichment analysis was performed for the list of candidate genes obtained from DIABLO discriminant analysis and for each candidate DcoExp module. For GO terms, the function pairwise_termsim () from enrichplot was used to calculate the Jaccard correlation coefficient among terms, resulting in a similarity matrix. Subsequently, the terms were functionally grouped using the similarity matrix to identify classes of closely related terms and reduce redundancy across terms. For the functional grouping, all annotated GO terms were included to estimate the similarity matrix, disregarding the enrichment status.

**FIGURE 1:**
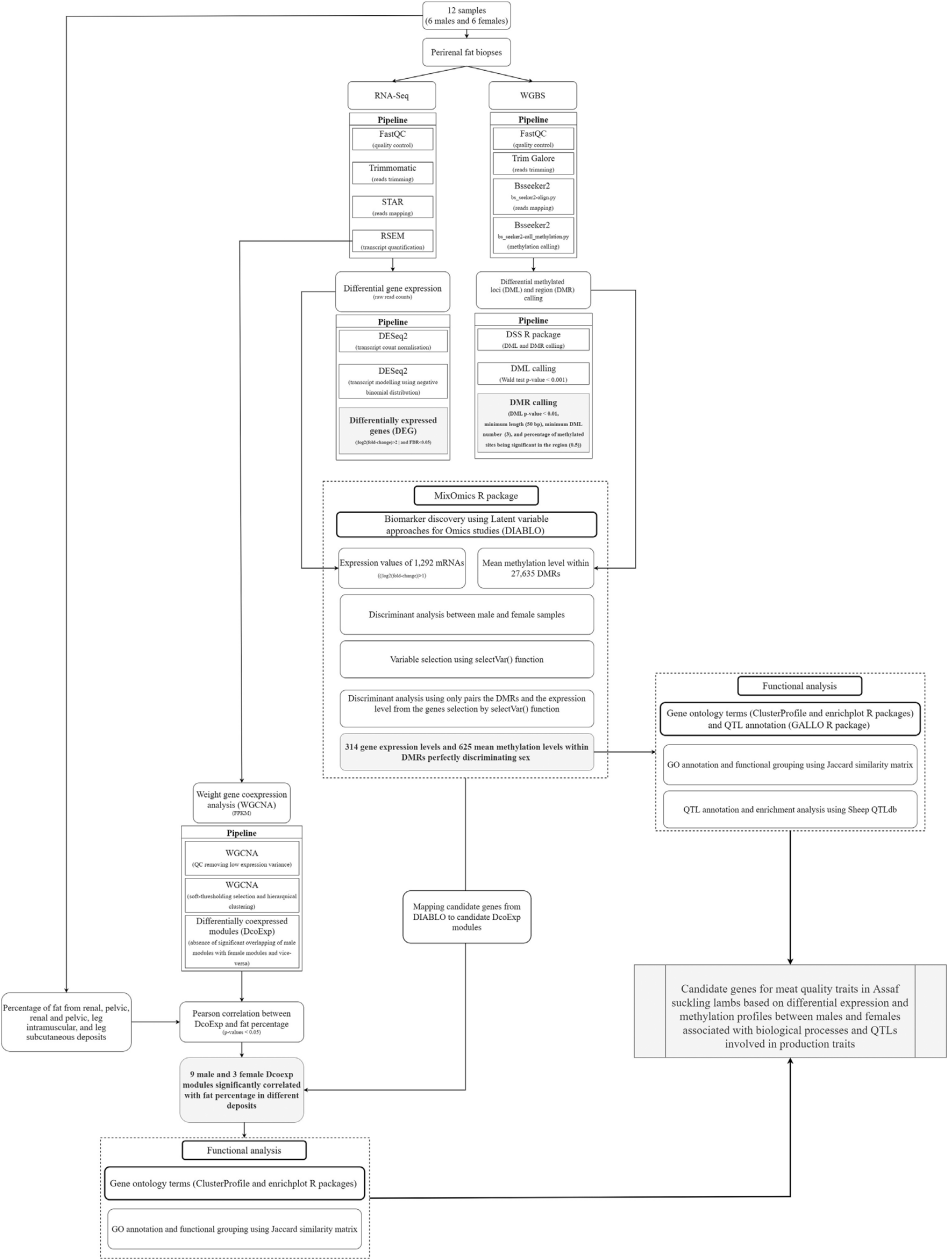
Flowchart summarizing the methods applied and the respective objectives reached in the current study. The grey boxes highlight the main results obtained in the methodological pipeline designed and applied in the present study.

Figure 1 shows a flowchart summarizing the methodology applied and described here.

## Results

### Phenotypic comparison between male and female samples

The mean values of the percentage of perirenal, subcutaneous and intramuscular fat for males and females are presented in Supplementary Table S1. There was no significant difference in age between male and female samples (*p* value > 0.05). The Anderson‒Darling test indicated that the percentage of renal (*p* value = 0.403), pelvic (*p* value = 0.483), renal and pelvic (*p* value = 0.575), leg intramuscular (*p* value = 0.403), and leg subcutaneous (*p* value = 0.560) fat followed a normal distribution. Additionally, the F test for equity of variance indicated an equal variance between males and females for percentage of renal (*p* value = 0.950), pelvic (*p* value = 0.399), renal and pelvic (*p* value = 0.404), leg intramuscular (*p* value = 0.661), and leg subcutaneous (*p* value = 0.234 fat. Significant differences (*p* value <0.05) were observed only for cavitary fat between males and females, and males had a higher percentage of pelvic fatness (Supplementary Table S1).

### Differentially expressed genes between males and females

An average of 23.695 (±1.038) million reads were obtained during the RNA-Seq analysis, from which, on average, 93.101% (±0.408%) were uniquely mapped to the ovine reference genome (Supplementary Table S2). A total of four DEGs were identified between males and females in the perirenal fat (Table 1). Among these four genes, two had an assigned gene symbol (*GPR143* and *CDH20*), and two were lncRNAs (*LOC101112291* and *LOC121817091*). Interestingly, *LOC101112291* is a lncRNA characterised as the XIST (X inactive specific transcript) orthologue in sheep. The two most DEGs were mapped to the ovine X chromosome (*LOC101112291* and *GPR143*), while the other two genes were mapped to chromosomes 23 (*CDH20*) and 18 (*LOC121817091*). Although few genes were differentially expressed based on the defined threshold, 1,292 genes showed an |log_2_ (fold-change)>1|, indicating potential coexpression differences between males and females (Supplementary Table S3).

**TABLE 1:** Differentially expressed genes (|log_2_(fold-change)|>2 and FDR<0.05) between males and females in the perirenal fat of Assaf suckling lambs.

Gene	CHR	log_2_(FC)	lfcSE	*p*-value	FDR
*LOC101112291*	X	14.754	0.872	3.30 × 10^−64^	5.86 × 10^−60^
*GPR143*	X	−3.280	0.567	7.34 × 10^−9^	6.53 × 10^−5^
*CDH20*	23	−6.914	1.495	3.78 × 10^−6^	2.24 × 10^−2^
*LOC121817091*	18	−4.006	0.906	9.79 × 10^−6^	4.35 × 10^−2^

CHR, chromosome; FC, fold change; lfcSE, log2(fold-change) standard error; FDR, false discovery rate.

### Differentially coexpressed modules of genes between males and females

All the samples and 6.928 genes were retained after the QC was performed for the WGCNA analysis. In males and females, 94 and 111 coexpressed modules were identified (Supplementary Figure S1). The pairwise comparison between males and females identified 35 and 27 coexpressed modules with a specific coexpression pattern (FDR<0.05) in each sex. The genes assigned to each DcoExp module are shown in Supplementary Table S4.

### Differential methylated regions between males and females

**FIGURE 2:**
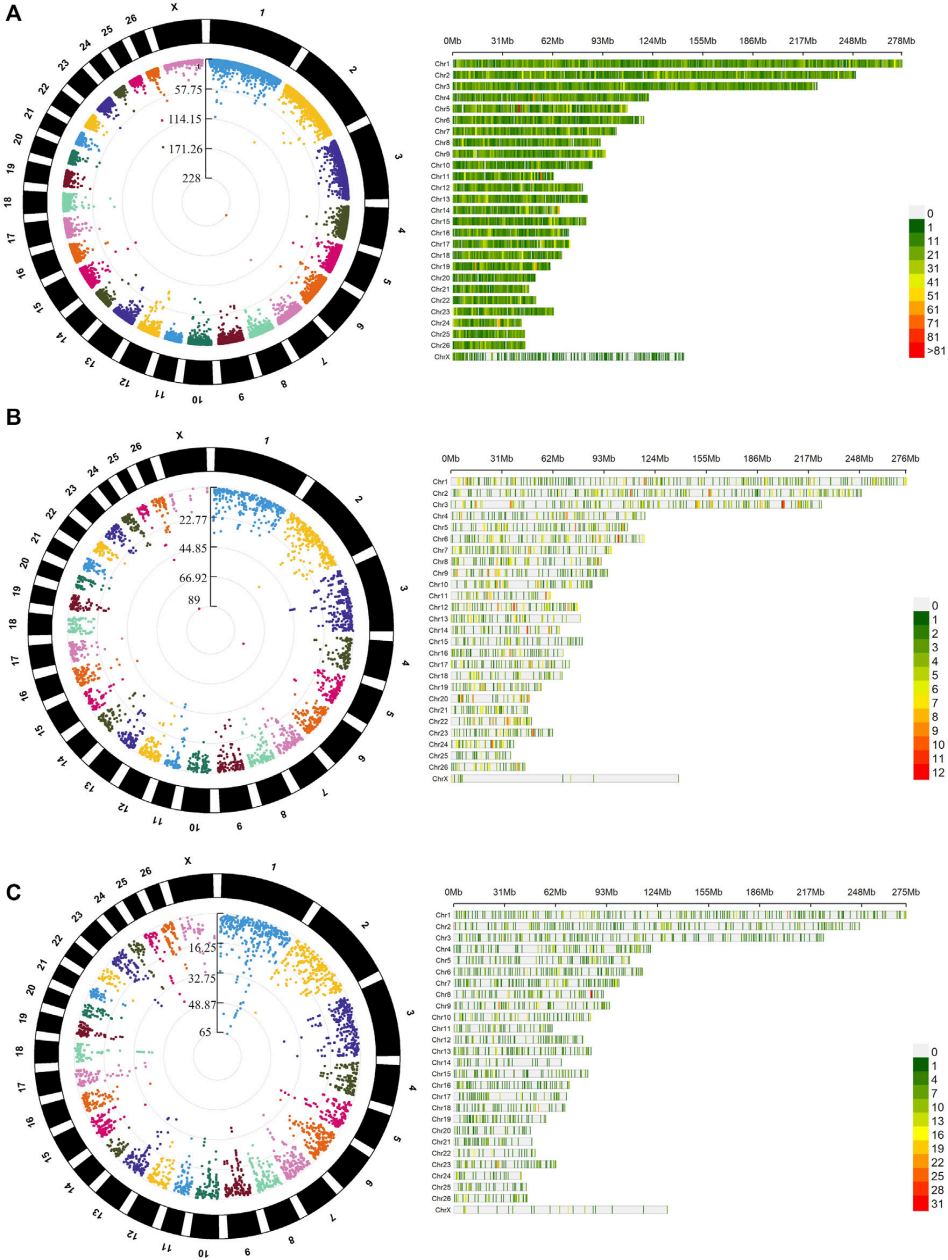
Distribution and genomic density of differentially methylated loci (DMLs) across the genome of Assaf lambs in the three evaluated methylation contexts CG **(A)**, CHG **(B)**, and CHH **(C)**. On the right-hand side, the circular Manhattan plots show the distribution of–DMLs in the comparison between males and females (log_10_ (*p*-values) after false discovery rate adjustment for the detected). On the left-hand side, the bar plots show the density of DMLs within 1 Mb windows for each sheep chromosome. A darker red shade indicates a higher number of DMLs within the 1 Mb windows.

**FIGURE 3:**
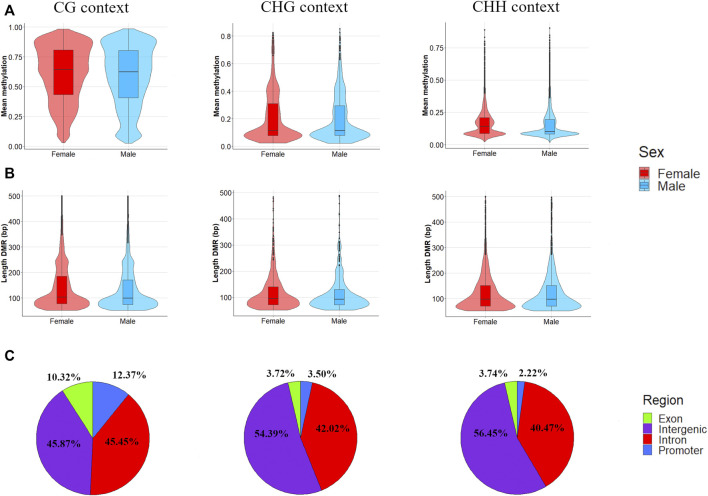
Distribution of **(A)** mean methylation level **(B)** length, and **(C)** genomic context (exon, intergenic, intron or promoter region) for the differential methylation regions (DMRs) detected between males and females in Assaf suckling lambs. **(A)** Violin plots showing the distribution of mean methylation levels within DRMs in the three methylation contexts (CG, CHG and CHH) and between males (in blue) and females (in red). **(B)** Violin plots showing the distribution of the DMR lengths in the three methylation contexts (CG, CHG and CHH) and between males (in blue) and females (in red). The length of DMRs was truncated at 500 bp for all three contexts to obtain a clear visualization of the distributions. **(C)** Pie plots showing the percentage of DMRs detected within each genomic context (intergenic, exon, intron and promoter).

The average mapping statistics for the WGBS data for all the samples are available in Supplementary Table S5. An average mapping rate of 70.46% (±1.21%) was obtained for the 12 samples analysed. The analysis of differential methylation between males and females identified 49,314 DMLs (CG = 40,158, CHG = 3,787, CHH = 5,369) and 27,635 DMRs (CG = 20,185, CHG = 1,342, CHH = 6,108), as shown in Supplementary Tables S6, S7. In Figure 2, circular Manhattan plots and density plots showing the distribution of significant DMLs across the ovine genome are shown for each methylation context (CG, CHG and CHH). As expected, higher methylation means were obtained for the DMRs mapped within CG contexts (males = 0.587 ± 0.252, females = 0.608 ± 0.234) when compared with CHG (males = 0.198 ± 0.168, females = 0.206 ± 0.175) and CHH (males = 0.155 ± 0.117, females = 0.167 ± 0.113) contexts. The comparison between males and females regarding the distribution of methylation means showed similar kernel densities in the three contexts (Figure 3A). The Cohen’s D obtained for the comparison between males and females in CG (0.085), CHG (0.044), and CHG (0.108) contexts corroborates this result, indicating a small sex effect for the global methylation pattern. Despite this small effect over the global methylation, interesting site-specific differences were observed for the identified DMRs, which will be presented below. Regarding the comparison of DMR lengths across the three different contexts, similar distributions were obtained for CG (251.5 ± 472.4), CHG (137.7 ± 210.01) and CHH (143.6 ± 186.10), as shown in Figure 3B. The Cohen’s D values obtained for the comparisons of DMR lengths between CG vs CHG (0.247), CG vs CHH (0.254), and CHG vs CHH (0.031) suggest small effects of the methylation context on the length of DMRs. However, higher effects were observed for the comparisons, including the DMRs within the CG context compared to CHG vs CHH, indicating longer DRMs. Regarding the comparison of different genomic contexts (intergenic, promoter, intron and exon) between the three methylation contexts, the CG context showed a higher percentage of DMRs mapped in promoters and exons (12.37% and 10.32%) than the other contexts (Figure 3C).

Among the identified DMRs, four were mapped within the coordinates of DEGs (Table 2). The first DMR (X:66088104–66090171) was mapped to *LOC101112291 (Xist lncRNA),* and the DMLs mapped within this DMR had a mean methylation level of 0.934 in males and 0.367 in females. The other two DMRs are mapped within the coordinates of *CDH20* and have contrasting methylation levels between each other. The DMR located on 23:60160365–60160625 has higher methylation in females (mean methylation females = 0.846, mean methylation in males = 0.528), while the DMR located on 23:60216480–60216888 has higher methylation in males (mean methylation females = 0.611, mean methylation in males = 0.821). The last DMR maps within the coordinates of *GPR143* show higher mean methylation in males (0.253) than in females (0.076).

**TABLE 2:** Statistics from the Differentially methylated regions (DMRs) identified within the coordinates of differentially expressed genes between male and female perirenal fat of Assaf suckling lambs. For each DMR, the location in the reference genome (chromosome: coordinates), the annotated gene in that region, the length in bp, the number of methylated cytosines, the proportion of methylation in females and males, and the sequence and genomic context are indicated.

DMR coordinates	Gene	Length (bp)	Number of methylated C	Mean methylation in females	Mean methylation in males	Context	Genomic context
X:66088104–66090171	*LOC101112291*	2068	4	0.367	0.934	CG	Exon/Intron
X:483776–483832	*GPR143*	57	11	0.076	0.253	CHH	Intron
23:60160365–60160625	*CDH20*	261	10	0.846	0.528	CG	Intergenic
23:60216480–60216888	*CDH20*	409	5	0.611	0.821	CG	Intron

DMR, differential methylated regions; bp, base pair.

### Discriminant analysis between males and females by multi-Omics integration

**FIGURE 4:**
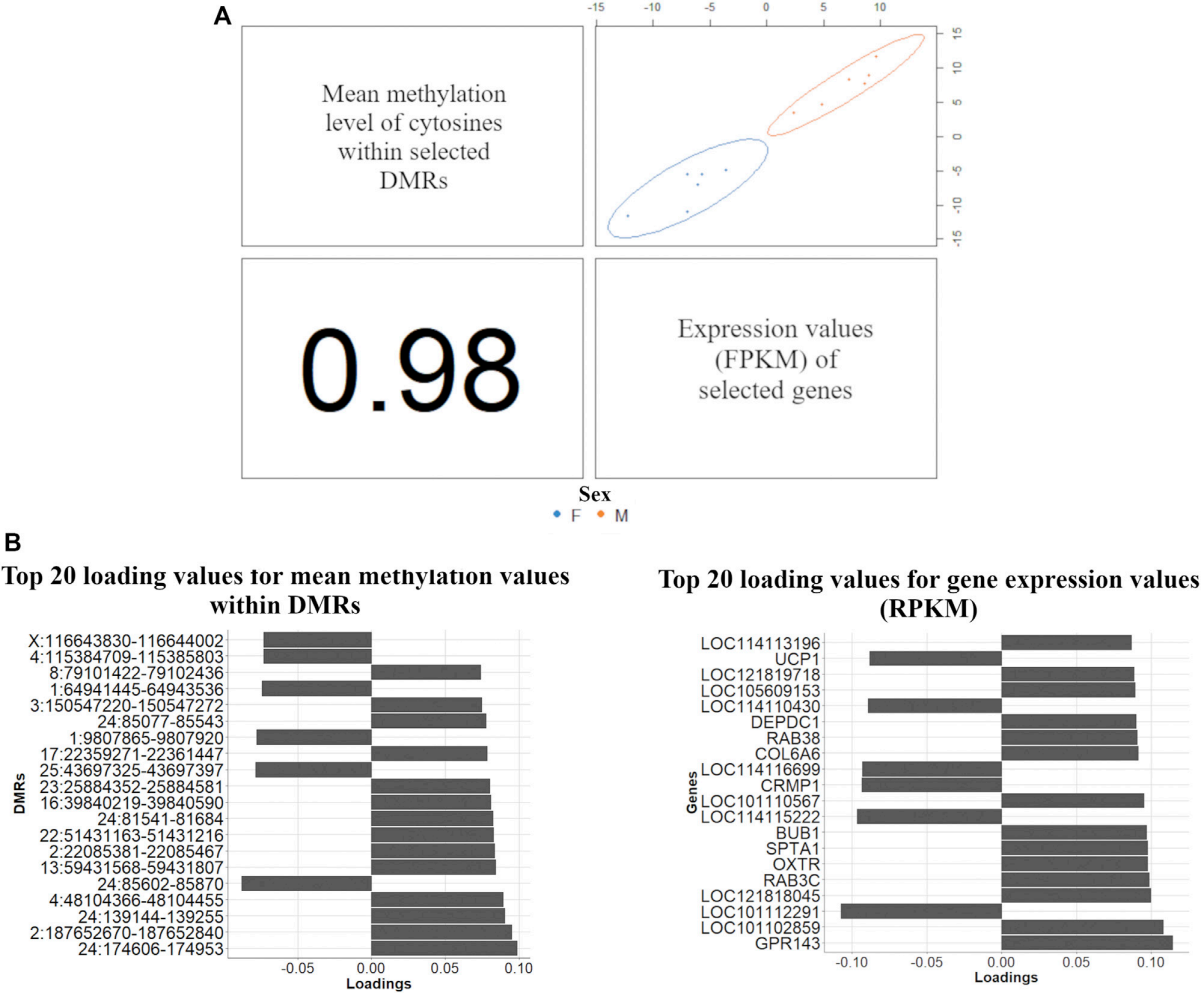
Multi-omics discriminant analysis between males (blue) and females (orange) samples using multiblock (s)PLS-DA. **(A)** Scatterplot from plotDiablo displaying the first principal component in RNA-Seq and WGBS datasets (upper diagonal plot), thus, constructed with expression values of 314 discriminant genes between males and females (RNA-Seq data) and 627 mean methylation levels within DMRs mapped within the same genes (WGBS data), and Pearson correlation (0.98) between each component (lower diagonal plot) **(B)** Loading values for the top 20 DMRs (left-hand side) and gene expression values (right-hand side) obtained in the multiblock (s)PLS-DA used to discriminate male and female samples.

The DIABLO discriminant analysis generated one prediction per dataset: 1,292 mRNAs (|log2 (fold-change)|>1) with the expression levels in FPKM and the mean methylation level of the cytosines within the DMRs. The variable selection procedure included all the genes and 27,635 mean methylation levels within DMRs in the list of selected variables. An AUC = 1 (*p* value = 0.004) was obtained for this selected dataset (for both DMRs and gene expression in the two principal components), consequently perfectly classifying the samples between males and females. Subsequently, the discriminant analysis was performed again using the expression levels of 314 genes due to overlapping between the selected mRNA expression levels and the methylation levels of cytosines within DMRs. A total of 627 DMRs were mapped within the coordinates of these 314 genes (204, 101, and 302 within CG, CHG and CHH contexts, respectively). The DIABLO discriminant analysis exclusively using the 314 genes again resulted in an AUC = 1 (*p* value = 0.0039) for both datasets and using the two first principal components for the discriminant analysis. The loading vectors for all tested variables in the two principal components are shown in Supplementary Table S8. The components used to discriminate male and female samples from selected mRNA expression levels and the methylation levels of cytosines within DMRs showed a correlation of 0.98 (Figure 4).

**FIGURE 5:**
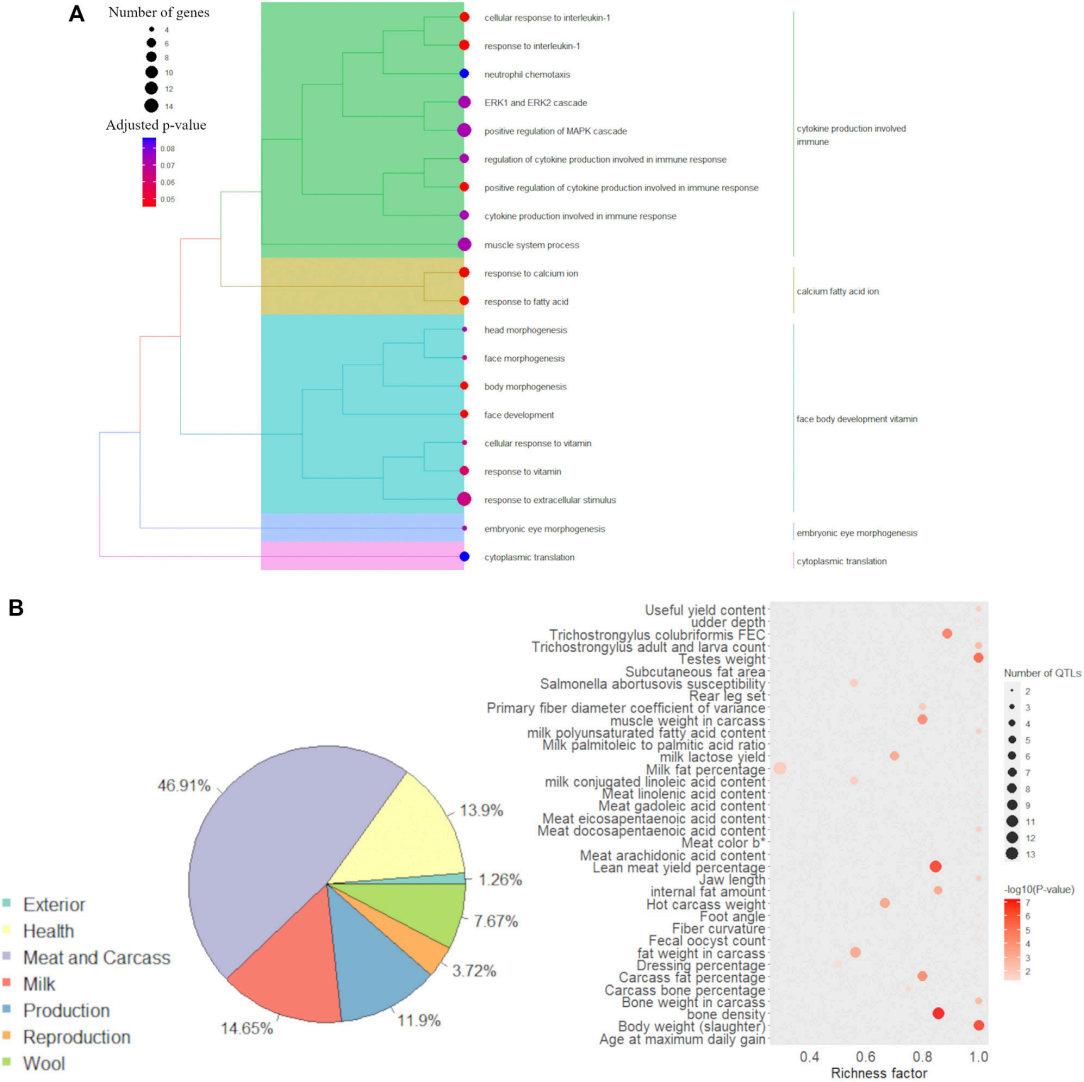
Functional analysis for the 314 discriminant genes obtained in the (s)PLS-DA discriminant analysis between male and female samples. **(A)** Functional grouping tree diagram for the Gene Ontology (GO) terms annotated for the 314 genes selected after the multiblock (s)PLS-DA discriminant analysis. Each color in the dendrogram represents a functional group obtained after estimating the Jaccard correlation coefficient. The area of the circles represents the number of genes assigned to each GO term, and the color of the circle indicates the *p*-value estimated for each GO term. **(B)** Pie plot showing the percentage of each QTL type annotated within the genomic coordinate of the 314 discriminant genes and bubble plot with QTL enrichment analysis results, where the darker the shade of red, the smallest is the enrichment *p*-value and the area of the circle represents the number of annotated QTLs.

The results of the annotation of GO terms for the list of 314 genes selected in the DIABLO discriminant analysis are shown in Supplementary Table S9. The redundancy reduction through the Jaccard correlation coefficient resulted in interesting groups of GO terms associated with the 314 selected genes (Figure 5A). The major GO term group, i.e., the term grouping the highest number of significant GO terms, was “cytokine production involved immune”. Additionally, the groups of GO terms associated with “calcium fatty acid ion”, “face body development vitamin”, “embryonic eye development” and “cytoplasmic translation” were also observed to be associated with the 314 selected genes.

The annotation of QTLs (previously reported in the literature) within the genomic coordinates of the 314 genes selected from the DIABLO discriminant analysis showed that the largest proportion of reported QTLs in those regions were associated with meat and carcass QTLs (46.91%). Figure 5B shows the percentage of all QTL types previously described within the genomic coordinate of the candidate genes. The complete list of annotated QTLs is available in Supplementary Table S10. The QTL enrichment analysis indicated the enrichment of traits associated with several QTL types available on the sheep QTL database (Figure 5B). However, it is interesting to highlight the presence of traits related to the total amount of fat, such as “age at maximum daily gain”, “body weight (slaughter)”, “carcass fat percentage”, “fat weight in carcass”, “internal fat amount”, “lean meat yield percentage”, and “subcutaneous fat area”. Additionally, QTLs associated with the content of different fatty acids in the meat were identified as enriched, such as for the following fatty acids: docosapentaenoic, eicosapentaenoic, gadoleic, linoleic, and arachidonic.

### Candidate DcoExp module of genes between males and females potentially associated with production traits

**FIGURE 6:**
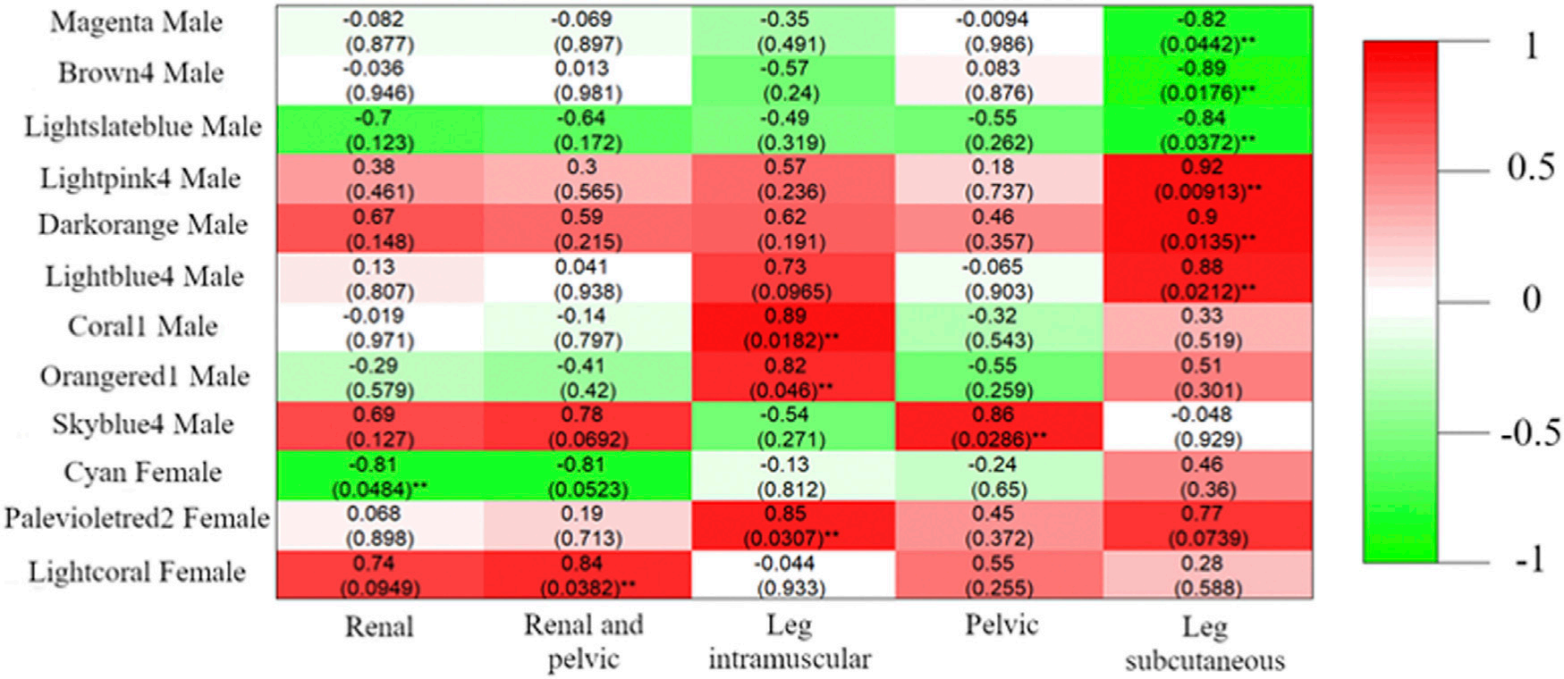
Pearson correlation between module eigengenes and fat percentage in different adipose deposits (Renal, Pelvic, Renal and Pelvic, Leg subcutaneous, and Leg intramuscular). The modules shown in the figure are the differentially coxpressed modules harboring at least one of the 314 discriminant genes between males and females. For each module, after the color identification of the module, it is indicated if the coexpressed gene module was identified in males or females. The Pearson correlation between the fat percentage and the module is shown in the figure for each specific fat deposit, and the *p*-value assigned for each correlation coefficient is shown between parenthesis (^**^ *p*-value<0.05). The color scale represents the signal of the correlation coefficient, where green shades represent negative correlations and red shades positive correlations.

In total, nine male DcoExp and three female DcoExp were significantly associated with the fat percentage in at least one of the fat deposits evaluated (Figure 6). Positive correlations were observed between the percentage of subcutaneous fat in the leg and the male DcoExp modules lightpink (*r* ^2^ = 0.92), darkorange (*r* ^2^ = 0.90) and lightblue (*r* ^2^ = 0.88). In contrast, a negative correlation was observed with the male brown4 module (*r* ^2^ = -0.89), magenta (*r* ^2^ = -0.82) and lightslateblue (*r* ^2^ = -0.84). Similarly, two male DcoExp modules, lightblue4 (*r* ^2^ = 0.89) and skyblue4 (*r* ^2^ = 0.82), were positively correlated with the percentage of intramuscular fat in the leg. Additionally, the male DcoExp, skyblue4, was positively correlated with the percentage of pelvic fat (*r* ^2^ = 0.86). Regarding the female DcoExp modules, the cyan female module was negatively correlated with the percentage of renal fat (*r* ^2^ = -0.81), while the lightcoral female module was positively correlated with the percentage of renal and pelvic fat (*r* ^2^ = 0.84). In addition, the palevioletred2 female module was positively correlated with the percentage of intramuscular fat in the leg (*r* ^2^ = 0.85).

Interestingly, all the DcoExp modules showing significant correlations with the fat percentage in the different fat deposits evaluated harboured at least one of the genes selected through the DIABLO discriminant analysis (Table 3). The top 10 enriched GO terms for each DcoExp module selected (when more than 10 terms were enriched) are shown in Table 4. Additionally, all of the associated GO terms are available in Supplementary Table S11. The analysis of redundancy reduction through the similarity matrix calculated using the Jaccard correlation coefficient resulted in identifying interesting biological processes associated with the selected DcoExp modules (Supplementary Figure S2).

**TABLE 3:** Sex discriminant genes identified by the multiblock (s)PLS-DA* discriminant analysis. These genes were allocated in differentially coexpressed modules from the weighted correlation network analysis (WGCNA)*, and were significantly correlated with the fat percentage in different adipose deposits (Renal, Pelvic, Renal and Pelvic, Leg subcutaneous, and Leg intramuscular). For each gene is indicated: their symbol, the description, the coexpression module, the fold change (base 2 logarithm), the DMR location in the reference genome (chromosome: coordinates), the proportion of methylation in females and males, and the sequence and genomic context.

Gene	Description	Module WGCNA^a^	log_2_(FC)^a^	DMR coordinate	Mean female methylation	Mean male methylation	Nucleotide context	Genomic context
*COL6A6*	Collagen type VI alpha 6 chain	Cyan-Female	−1.134	1:272857035–272858265	0.511	0.934	CG	Intron
*BUB1*	BUB1 mitotic checkpoint serine/threonine kinase	Cyan-Female	−1.185	3:104905951–104906039	0.203	0.068	CG	Promoter
				3:104906340–104906756	0.413	0.076	CG	Promoter
*SKA1*	Spindle and kinetochore associated complex subunit 1	Cyan-Female	−1.182	23:50315601–50315664	0.712	0.172	CHH	Intron
*LOC121819057*	Uncharacterized LOC121819057	Cyan-Female	1.147	3:79227975–79228136	0.086	0.230	CHH	Intergenic
				3:79164130–79164185	0.560	0.340	CG	Intergenic
*LOC101113771*	C-C motif chemokine 3-like	Lightcoral-Female	1.235	11:13965372–13965500	0.504	0.895	CG	Intron
*LOC114113921*	KATNB1-like protein 1	Lightcoral-Female	1.004	3:53093129–53093208	0.764	0.905	CG	Intergenic
				3:53428831–53431256	0.423	0.526	CG	Intergenic
				3:53749953–53750047	0.908	0.328	CG	Intergenic
				3:53914562–53914855	0.802	0.575	CG	Intergenic
				3: 53536355–53536428	0.179	0.527	CHH	Intergenic
				3: 53263175–53263232	0.227	0.081	CHH	Intergenic
				3: 53338513–53338574	0.270	0.102	CHH	Intergenic
*LOC105605978*	Tumor necrosis factor receptor superfamily member 26-like	Palevioletred2-Female	−1.236	21: 44932535–44932634	0.140	0.080	CHG	Intron
*PLA2G5*	Phospholipase A2 group V	Brown4-Male	−1.064	2:246336958–246337395	0.528	0.909	CG	Intergenic
				2:246357555–246357847	0.811	0.484	CG	Intron
*BCL2L14*	BCL2 like 14	Darkorange-Male	−1.227	3:203381087–203381150	0.906	0.653	CG	Intergenic
				3:203398094–203398169	0.152	0.631	CG	Intron
				3:203402162–203402245	0.225	0.715	CG	Intron
				3:203370171–203370245	0.254	0.100	CHH	Intergenic
*CLEC2D*	C-type lectin domain family 2-member D	Darkorange-Male	1.403	3:206065407–206065635	0.351	0.642	CG	Intergenic
				3:206089993–206090043	0.149	0.086	CHH	Intron
*VSTM5*	V-set and transmembrane domain containing 5	Darkorange-Male	−1.130	21:561263–561315	0.245	0.094	CHH	Intron
				21:472974–473091	0.396	0.175	CG	Intergenic
*BFSP2*	Beaded filament structural protein 2	Lightblue4-Male	−1.522	1:256647462–256647584	0.248	0.452	CG	Intron
				1:256784172–256784621	0.932	0.490	CG	Intron
				1:256737014–256737067	0.226	0.083	CHG	Intron
*LOC101107700*	Uncharacterized protein C4orf19-like	Lightblue4-Male	1.549	23:40021734–40021852	0.575	0.338	CG	Intergenic
				23:40128687–40129173	0.609	0.889	CG	Intergenic
				23:40143977–40144111	0.206	0.081	CHH	Intergenic
*BUB1*	BUB1 mitotic checkpoint Serine/threonine kinase	Lightslateblue-Male	−1.185	3:104905951–104906039	0.203	0.068	CG	Promoter
				3:104906340–104906756	0.413	0.076	CG	Promoter
*LOC114111438*	Uncharacterized LOC114111438	Lightslateblue-Male	−1.021	X:99973590–99973672	0.720	0.830	CG	Exon
				X:99975373–99975613	0.053	0.092	CHG	Exon
*EMX2*	Empty spiracles homeobox 2	Skyblue4-Male	−1.021	22:37376592–37376681	0.374	0.826	CG	Intergenic
				22:37386811–37386967	0.797	0.417	CG	Intergenic
				22:37429072–37429186	0.942	0.795	CG	Intergenic
*ARG2*	Arginase 2	Skyblue4-Male	1.428	7:77567672–77567760	0.172	0.077	CHH	Intron
*LOC101120997*	Anaphase-promoting complex subunit 15-like	Skyblue4-Male	1.167	3:110380188.110380337	0.695	0.295	CHG	Intergenic
				3:110440297.110440384	0.841	0.547	CG	Intergenic
				3:110442593.110442698	0.710	0.930	CG	Intergenic
*RASSF5*	Ras association domain family member 5	Coral1-Male	1.085	12:44932535–44932634	0.141	0.321	CG	Intergenic
				12:44932535–44932634	0.110	0.069	CHH	Intron
*LOC114112700*	Translation initiation factor IF-2-like	Magenta-Male	1.008	2:250131883–250132043	0.067	0.050	CHG	Promoter/Exon
				2:250132404–250132465	0.082	0.061	CHG	Promoter/Exon
				2:250139818–250139925	0.047	0.029	CHG	Intergenic
				2:250148659–250148782	0.075	0.058	CHH	Intergenic
				2:250139850–250140333	0.046	0.031	CHH	Intergenic
				2:250139994–250140087	0.047	0.030	CHG	Intergenic
				2:250149933–250150397	0.225	0.140	CHH	Intergenic
*LOC114112008* *^a^*	Liprin-alpha-1-like	Orangered1-Male	−2.420	24:206621–206706	0.462	0.701	CG	Exon/Intron
				24:163454–163594	0.676	0.807	CG	Intergenic
				24:81541–81684	0.100	0.052	CHG	Intergenic
				24:81740–81805	0.095	0.050	CHG	Intergenic
				24:154547–155044	0.112	0.068	CHH	Intergenic
*LOC121818175*	Small integral membrane protein 13-like	Orangered1-Male	−1.217	X:79759026–79759116	0.101	0.516	CHH	Intergenic
				X:79759026–79759116	0.101	0.516	CHH	Intergenic
*LOC121818524*	Collagen alpha-1(I) chain-like	Lightpink4-Male	1.115	2: 250122227–250122458	0.062	0.047	CHG	Intergenic

^a^ Only the top five DMRs, with the highest absolute methylation mean between males and females are shown for the gene LOC114112008, as 74 DMRs, were annotated within the coordinates of this gene.

**TABLE 4:** Top ten enriched (FDR< 0.05) gene ontology terms (when available) for genes allocated within the differentially coexpressed modules between males and females Assaf suckling lambs correlated with the fat percentage in different fat deposits (Renal, Pelvic, Renal and Pelvic, Leg subcutaneous, and Leg intramuscular). For each module, the gene ontology process (see table footnote), its specific description, the nominal *p*-value, the adjusted FDR *p*-value, and the genes involved in the GO term are indicated.

Module	Gene ontology*	Description	*p*-value	FDR	Gene symbol
Brown4-Male	CC	ISGF3 Complex	6.81 × 10^−05^	0.014	*STAT1, STAT2*
	BP	Response to Virus	1.47 × 10^−05^	0.026	*CCDC92, HYAL1, IFI44, IFIT2, TRIM5, RNASEL, SAMHD1, STAT1, STAT2, TNFSF4*
	CC	HOPS Complex	4.70 × 10^−04^	0.048	*VPS18, VPS33B*
Darkorange-Male	CC	Spindle Pole	5.65 × 10^−05^	0.022	*BIRC6, CEP128, CUL3, DYNC1LI1, GPSM2, CALM1, NDE1, NIN, SPDL1, TOPBP1*
	MF	Adenylylsulfate Kinase Activity	1.74 × 10^−04^	0.027	*PAPSS1, PAPSS2*
	MF	Sulfate Adenylyltransferase Activity	1.74 × 10^−04^	0.027	*PAPSS1, PAPSS2*
	MF	Sulfate Adenylyltransferase (ATP) Activity	1.74 × 10^−04^	0.027	*PAPSS1, PAPSS2*
	MF	2-Acylglycerol-3-Phosphate O-Acyltransferase Activity	1.82 × 10^−04^	0.027	*LPCAT2, LPCAT4, LPGAT1*
	CC	Nem1-Spo7 Phosphatase Complex	1.56 × 10^−04^	0.031	*CNEP1R1, CTDNEP1*
	MF	Serine Transmembrane Transporter Activity	3.59 × 10^−04^	0.042	*SFXN1, SFXN3, SLC38A2*
Lightblue4-Male	CC	Mitochondrial Inner Membrane	9.84 × 10^−06^	0.001	*DNAJC30, ATAD3A, CYC1, SLC25A12, TIMM22, TIMM29, TIMMDC1, TMEM186*
	CC	Mitochondrial Matrix	6.94 × 10^−05^	0.003	*ATAD3A, METTL17, MMAB, PARS2, SDHAF2, TFB2M, TRUB2*
	CC	TIM22 Mitochondrial Import Inner Membrane Insertion Complex	9.81 × 10^−05^	0.003	*TIMM22, TIMM29*
	MF	Pseudouridine Synthase Activity	3.90 × 10^−04^	0.048	*RPUSD2, TRUB2*
	MF	Nuclear Steroid Receptor Activity	0.001	0.048	*GPER1, PGR*
	MF	Intramolecular Transferase Activity	0.001	0.048	*RPUSD2, TRUB2*
	MF	Protein Transporter Activity	0.002	0.048	*TIMM22, TIMM29*
	MF	N-Acetylglucosamine-6-Phosphate Deacetylase Activity	0.002	0.048	*AMDHD2*
	MF	Cob(I)yrinic Acid a,c-Diamide Adenosyltransferase Activity	0.002	0.048	*MMAB*
	MF	N6-Isopentenyladenosine Methylthiotransferase Activity	0.002	0.048	*CDK5RAP1*
Lightslateblue-Male	MF	3′-5′-Exoribonuclease Activity	8.19 × 10^−05^	0.021	*EXOSC3, EXOSC7, NOCT, PNPT1*
	MF	Exoribonuclease Activity, Producing 5′-Phosphomonoesters	1.14 × 10^−04^	0.021	*EXOSC3, EXOSC7, NOCT, PNPT1*
	MF	Exoribonuclease Activity	1.39 × 10^−04^	0.021	*EXOSC3, EXOSC7, NOCT, PNPT1*
	CC	Cytoplasmic Exosome (Rnase Complex)	7.42 × 10^−05^	0.021	*EXOSC3, EXOSC7, PNPT1*
	CC	Exosome (Rnase Complex)	2.44 × 10^−04^	0.027	*EXOSC3, EXOSC7, PNPT1*
	CC	Exoribonuclease Complex	2.86 × 10^−04^	0.027	*EXOSC3, EXOSC7, PNPT1*
	BP	Polyadenylation-Dependent RNA Catabolic Process	1.45 × 10^−05^	0.038	*EXOSC3, EXOSC7, PNPT1*
	CC	Specific Granule	6.45 × 10-^04^	0.046	*HPSE, VAMP8, BST1, PTPN6, SLC2A5, VAMP1*
	MF	3′-5′ Exonuclease Activity	4.49 × 10^−04^	0.049	*EXOSC3, EXOSC7, NOCT, PNPT1*
	MF	Exonuclease Activity, Active with Either Ribo- Or Deoxyribonucleic Acids and Producing 5′-Phosphomonoesters	6.30 × 10^−04^	0.049	*EXOSC3, EXOSC7, NOCT, PNPT1*
Skyblue4-Male	CC	Cytosolic Ribosome	2.32 × 10^−07^	4.92 × 10^−05^	*RPL13, RPS3A, RPL36A, RPS15A, RPL35A, RPL6, RPL37A*
	CC	Cytosolic Large Ribosomal Subunit	4.36 × 10^−06^	4.00 × 10^−03^	*RPL13, RPL36A, RPL35A, RPL6, RPL37A*
	CC	Ribosomal Subunit	9.71 × 10^−06^	6.86 × 10^−04^	*RPL13, RPS3A, RPL36A, RPS15A, RPL35A, RPL6, RPL37A*
	CC	Ribosome	4.90 × 10^−05^	0.002	*RPL13, RPS3A, RPL36A, RPS15A, RPL35A, RPL6, RPL37A*
	CC	Large Ribosomal Subunit	1.07 × 10^−04^	0.004	*RPL13, RPL36A, RPL35A, RPL6, RPL37A*
	BP	Cytoplasmic Translation	2.91 × 10^−06^	0.004	*RPL13, RPS3A, RPL36A, RPS15A, RPL35A, RPL6, RPL37A*
	MF	Structural Constituent of Ribosome	1.89 × 10^−05^	0.005	*RPL13, RPS3A, RPL36A, RPS15A, RPL35A, RPL6, RPL37A*
Orangered1-Male	BP	Response to insulin	1.18 × 10^−07^	2.27 × 10^−04^	*AKT2, CPEB2, CRY2, GHSR, KLF15, SLC2A4, SORBS1, SREBF1, TRIB3, VWA2, WDTC1*
	BP	Cellular response to insulin stimulus	1.35 × 10^−06^	9.023 × 10^−04^	*AKT2, CPEB2, GHSR, SLC2A4, SORBS1, SREBF1, TRIB3, VWA2, WDTC1*
	BP	Response to peptide hormone	1.41 × 10^−06^	9.023 × 10^−04^	*AKT2, CPEB2, CRY2, GHSR, KLF15, LTA4H, SLC2A4, SORBS1, SREBF1, TRIB3, VWA2, WDTC1*
	BP	Response to peptide	1.04 × 10^−05^	4.97 × 10^−04^	*AKT2, CPEB2, CRY2, GHSR, KLF15, LTA4H, SLC2A4, SORBS1, SREBF1, TRIB3, VWA2, WDTC1*
	BP	Cellular response to peptide	2.06 × 10^−05^	7.88 × 10^−03^	*AKT2, CPEB2, GHSR, KLF15, SLC2A4, SORBS1, SREBF1, TRIB3, VWA2, WDTC1*
	BP	Cellular response to peptide hormone stimulus	2.50 × 10^−05^	0.008	*AKT2, CPEB2, GHSR, SLC2A4, SORBS1, SREBF1, TRIB3, VWA2, WDTC1*
	BP	Hexose metabolic process	4.19 × 10^−05^	0.011	*AKT2, DCXR, OMA1, PFKFB3, PFKFB4, PPP1R3B, SORBS1, WDTC1*
	BP	Base-excision repair, gap-filling	4.94 × 10^−05^	0.011	*LIG1, PARG, POLE*
	BP	Monosaccharide metabolic process	7.13 × 10^−05^	0.015	*AKT2, DCXR, OMA1, PFKFB3, PFKFB4, PPP1R3B, SORBS1, WDTC1*
	BP	Regulation of fatty acid metabolic process	1.25 × 10^−04^	0.023	*AKT2, GHSR, SREBF1, TRIB3, WDTC1*
Cyan-Female	BP	Chromosome Segregation	4.46 × 10^−08^	7.00 × 10^−05^	*BRIP1, BUB1, CCNE1, CENPW, DLGAP5, UBE2I, MEIOB, PRC1, SKA1, TOP2A, UBE2C*
	BP	Nuclear Division	5.37 × 10^−07^	4.21 × 10^−04^	*BRIP1, BUB1, CCNE1, DLGAP5, KIF11, UBE2I, MEIOB, PRC1, TDRD9, TOP2A, UBE2C*
	BP	Organelle Fission	1.43 × 10^−06^	7.50 × 10^−04^	*BRIP1, BUB1, CCNE1, DLGAP5, KIF11, UBE2I, MEIOB, PRC1, TDRD9, TOP2A, UBE2C*
	CC	Condensed Chromosome	2.87 × 10^−05^	2.98 × 10^−04^	*BUB1, CENPW, HMGB2, UBE2I, SETMAR, SKA1, TOP2A*
	CC	Chromosomal Region	3.82 × 10^−05^	2.98 × 10^−04^	*BUB1, CDK1, CENPW, ORC1, RPA1, SKA1, TOP2A, ZNF827*
	MF	NADPH Dehydrogenase (Quinone) Activity	1.26 × 10^−05^	3.27 × 10^−04^	*CBR4, NQ O 1*
	BP	Nuclear Chromosome Segregation	8.94 × 10^−06^	3.50 × 10^−04^	*BRIP1, BUB1, CCNE1, DLGAP5, MEIOB, PRC1, TOP2A, UBE2C*
	BP	Regulation of Cell Cycle Phase Transition	1.77 × 10^−05^	5.52 × 10^−04^	*BRIP1, BUB1, CDK1, CDKN2C, DLGAP5, MDM2, ORC1, SETMAR, UBE2C*
	BP	Meiotic Chromosome Segregation	2.52 × 10^−05^	5.52 × 10^−04^	*BRIP1, BUB1, CCNE1, MEIOB, TOP2A*
	BP	Chromosome Separation	2.65 × 10^−05^	5.52 × 10^−04^	*BUB1, DLGAP5, MEIOB, TOP2A, UBE2C*
Lightcoral-Female	MF	Type I Transforming Growth Factor Beta Receptor Binding	8.59 × 10^−06^	2.68 × 10^−04^	*ENG, SMAD6, SMAD7*
	MF	Transcription Regulator Inhibitor Activity	9.21 × 10^−05^	0.014	*MDFI, SMAD6, SMAD7*
	MF	Transforming Growth Factor Beta Receptor Binding	1.38 × 10^−04^	0.014	*ENG, SMAD6, SMAD7*
	BP	Cardiac Septum Morphogenesis	1.20 × 10^−05^	0.022	*ENG, MSX2, SMAD6, SMAD7, TBX3*
	CC	Heteromeric SMAD Protein Complex	4.53 × 10^−04^	0.040	*SMAD6, SMAD7*
	CC	SMAD Protein Complex	5.81 × 10^−04^	0.040	*SMAD6, SMAD7*
	CC	Postsynaptic Cytoskeleton	5.81 × 10^−04^	0.040	*ACTB, SPTBN2*
	MF	Adrenergic Receptor Activity	6.25 × 10^−04^	0.048	*ADRA2B, ADRB1*
Palevioletred2-Female	MF	delta24 (24–1) sterol reductase activity	0.0021	0.0490	*DHCR24*
	MF	UDP-N-acetylglucosamine 4-epimerase activity	0.0021	0.0490	*GALE*
	MF	UDP-glucose 4-epimerase activity	0.0021	0.0490	*GALE*
	MF	Arylformamidase activity	0.0021	0.0490	*AFMID*
	MF	Mevalonate kinase activity	0.0021	0.0490	*MVK*
	MF	delta24-sterol reductase activity	0.0021	0.0490	*DHCR24*

## Discussion

### Differential gene expression analysis

*LOC101112291* was the transcript with the highest differential expression between females and males in the perirenal fat (FDR = 5.86 × 10^−60^), showing higher expression in female samples (log_2_(Fold-Change) = 14.754). This locus encodes an orthologous gene in sheep for the human XIST gene, a crucial lncRNA acting in the X-chromosome activation and expression balance of X-linked genes between males and females (Payer and Lee, 2008). In humans, XIST is more highly expressed in female subcutaneous fat than in males and in the subcutaneous fat of cortisol-producing adenoma female patients than in controls (Wu et al., 2019, Wu et al., 2022). Interestingly, a sex-specific expression pattern exclusive to females was observed in the subcutaneous and visceral adipose tissues of ob/ob mice (Shinozaki et al., 2007). Additionally, this gene has been associated with brown adipose tissue. For instance, the knockdown of XIST in human perirenal and subcutaneous tissues resulted in an inhibition of differentiation of brown preadipocytes. On the other hand, its overexpression promoted the full differentiation of brown preadipocytes (Wu et al., 2022). In the same study, the results indicate that XIST acts through direct binding to C/EBPα and BAT activation, consequently combating high-fat diet-induced obesity (Wu et al., 2022). It is interesting to highlight that a DMR was identified within the coordinates of *LOC101112291,* showing higher methylation mean for the DMLs within this DMR in male samples. The other DEGs were *GPR143* (log_2_(Fold-Change) = -3.280 and FDR = 6.53 × 10^−5^), *CDH20* (log_2_(Fold-Change) = -4.006 and FDR = 4.35 × 10^−2^), and *LOC121817091* (log_2_(Fold-Change) = -4.006 and FDR = 4.35 × 10^−2^). The functions of *GPR143* are related with regulation of whole-body metabolism and adipose tissue function (*GPR143*) (Premont and Gainetdinov, 2007; Amisten et al., 2008). The *CDH20* encodes a member of a cadherin superfamily and it was previously associated with backfat thickness at 100 kg in pigs through a genome-wide association study (Zhang et al., 2021). To the best of our knowledge, no link between *LOC121817091* and fat tissue and/or sex differences was previously described in the literature.

It is interesting to highlight that in the current study, healthy animals in an early stage of post-natal development were compared. Here, a small number of DEG was observed between males and females. The same expression pattern between males and females was previously described in other species, such as humans and cattle, in adipocytes and other tissues (Seo et al., 2016; Anderson et al., 2020; Rey et al., 2021). Consequently, slighter differences in the expression pattern might be expected. However, despite the small number of DEG, it is impossible to disregard potential differences between sexes caused by other mechanisms such as differential co-expression patterns, post-transcriptional mechanisms and environmental response.

### Discriminant analysis between male and female samples using expression profiles and DMRs

The functional grouping of the GO terms associated with the 314 genes selected in the DIABLO analysis indicated the presence of biological processes related to cytokine production and response to interleukin, as well as response to fatty acids (Figure 4).

The BAT secretes several molecules responsible for regulating functions in several organs by autocrine, paracrine and endocrine actions (Villarroya et al., 2017). These molecules are called batokines, and their activities have been associated with protectivity against obesity and metabolic diseases (Lowell et al., 1993). Among the main batokines, interleukin-6 (*IL-6*) and adiponectin can be highlighted due to their functional relevance. *IL-6* is among the 314 genes selected from the discriminant analysis. Recently, the specific action of IL-6-type cytokine signalling in adipocytes has been associated with the development of obesity-associated insulin resistance and steatosis (Allen and Febbraio, 2010). Additionally, evidence suggests that adipocyte-specific IL-6 induces the release of free fatty acids and leptin through an insulin effect, subsequently affecting liver metabolism and pancreatic β-cell function (Wueest and Konrad, 2018). Even though adiponectin was not among the 314 genes selected in the discriminant analysis, the genes *C1QTNF3* and *IL1B* were associated with biological processes related to the regulation of adiponectin secretion.

The genes *AKR1C2*, *CD36*, *CPS1*, *PCSK1*, *PON1*, and *UCP1* were among the 314 genes selected in the discriminant analysis and were associated with the biological process “response to fatty acids”. Among these genes, it is important to highlight the key function of *UCP1* in the thermogenesis of BAT in lambs, showing a fast decrease in the mRNA levels after birth as well as the BAT (Clarke et al., 1997; Saely et al., 2011). Consequently, *UCP1* is a classical biomarker for BAT. Recently, our research group demonstrated that the expression of *UCP1* mRNA follows the percentage of multilocular adipocytes (another BAT marker) in Assaf suckling lambs (Suárez-Vega et al., 2018). Here, *UCP1*, despite not being differentially expressed between males and females, showed a log_2_(FC) of 2.17. This finding indicates a higher expression in female Assaf suckling lambs, which corroborates the literature (Rodríguez et al., 2001; Rodríguez-Cuenca et al., 2002; Oliver et al., 2007).

The QTL enrichment analysis performed using the QTLs previously reported within the genomic coordinates of the 314 selected genes suggested that the regions harbouring these genes were frequently reported to be associated with relevant fat-related traits. For example, the “lean meat yield percentage” and “body weight (slaughter)” were the most enriched QTLs in this analysis, with 11 out of the 13 and nine out of nine QTLs reported in the sheep QTL database mapped within the coordinates of the 314 selected genes, respectively. A total of 64 and 59 genes out the 314 genes selected in the discriminant analysis were mapped within QTLs for “lean meat yield percentage” and “body weight (slaughter)”, respectively. The presence of other QTLs, such as “fat weight carcass” and “subcutaneous fat area”, reinforce the recurrent association of these regions with the total amount of fat in the individual. Additionally, the annotation of QTLs associated with the content of FA in the meat highlights the potential of these genes to be involved with meat quality traits.

### Differentially coexpressed gene modules between male and female samples significantly correlated with the percentage of fat in different fat deposits

Interestingly, after the DIABLO discriminant analysis, 12 of the DcoExp modules (nine for males and three for females) harboured 22 genes from the list of selected genes (Table 3). In addition, these DcoExp modules were significantly correlated with the fat percentage in at least one of the evaluated fat deposits. Differentially from a single gene differential expression analysis, a co-expression analysis has the potential to identify alterations in biological processes between groups even when small differences are observed individually for each gene expression profile. Therefore, here the biological processes associated with the differentially co-expressed modules between males and females were used to better understand the potential role of the candidate genes harboring DMRs and selected in the DIABLO analysis over meat quality traits.

It is important to highlight that results from DMRs and expression patterns must be interpreted carefully. The presence of a DMR near or within a gene coordinate not necessarily implicates in a differential expression pattern. A DMR must effectively change the accessibility of the transcriptional machinery to the DNA to change the expression pattern of a gene. In addition, it is interesting to mention that, for example, the same gene might have two or more DMRs with contrasting methylation patterns, which makes the interpretability even more complex. Therefore, this effective potential to module the expression profile should be validated using other techniques, such as ATAC-seq (Luo et al., 2022).

Enriched GO terms were not identified for the male DcoExp modules coral1, magenta, and lightpink4. The 314 selected genes from the discriminant analysis list and allocated within these modules were *RASSF5*, *LOC114112700*, and *LOC121818524*, respectively. *RASSF5*, also known as *NORE1A*, acts as an effector of the Ras protein (Donninger et al., 2016). The RAS protein is a strong activator of the ERK pathway, which is suggested to play a positive role in adipogenesis (Bost et al., 2005). *LOC114112700* is reported to be an orthologue of the translation initiation factor (IF-2) gene. However, no direct link between IF-2 and adipocytes was found. *LOC121818524* encodes an orthologue of the collagen alpha-1(I) chain (*COL1A1*) gene. *COL1A1* is a major component of the extracellular matrix in adipose tissue and is significantly suppressed by adipogenesis induction (Okada et al., 2012). Additionally, the expression of *COL1A1* and other collagen family members was low in beef cattle showing high marbling in the *longissimus dorsi* muscle (Chen et al., 2019).

The male module brown4 harboured the gene phosphatidylcholine 2-acylhydrolase 5 (*PLA2G5*), a member of the secretory phospholipase A2 family. *PLA2G5* protects against diet-induced obesity and insulin resistance with an additional function in the translation of macrophages from adipose tissue from the M1 to M2 state (Sato et al., 2014). In addition, *PLA2G5* knockout mice have hyperlipidaemia, increased obesity, hepatic steatosis, lower insulin sensitivity, greater infiltration of M1 macrophages, and a higher expression of proinflammatory cytokines (Sato et al., 2014).

In the lightblue4 male module are the genes Beaded filament structural protein 2 (*BFSP2*) and *LOC101107700* (uncharacterised protein C4orf19-like). To the best of our knowledge, there is not a direct association between these genes and adipocyte-related biological processes.

The darkorange module, DCoExp in males, harboured the genes Apoptosis Facilitator Bcl-2-Like Protein 14 (*BCL2L14*) and C-Type Lectin Domain Family 2 Member D (*CLEC2D*). *BCL2L14* is a proapoptotic member of the bcl-2 family, which seems to be associated with an important role in the GSK3β-mediated osteoblast apoptosis process (Guo et al., 2001; Nie et al., 2018). However, no direct link between *BCL2L14* and biological processes associated with lipid metabolism were identified.

The male modules lightslateblue and skyblue4 harboured the genes BUB1 mitotic checkpoint serine/threonine kinase (*BUB1*) and Empty spiracles homeobox 2 (*EMX2*), respectively. Interestingly, the dysregulation of BUB1 signalling drives increased proliferation of lipoedema in adipose-derived stem cells, which suggests a potential role in the regulation of adipogenesis (Ishaq et al., 2022). The *EMEX2* gene is a homeobox gene that encodes a transcription factor initially associated with cerebral development (Cecchi, 2002). In humans, *EMX2* was previously identified as differentially expressed in subcutaneous fat *versus* visceral adipose tissue, subcutaneous fat *versus* omental preadipocytes, and subcutaneous *versus* perirenal and perivascular adipocytes (Tchkonia et al., 2007; Omar et al., 2014; Keller et al., 2017). Interestingly, *EMX2* was identified as upregulated in abdominal subcutaneous tissue after fat loss in humans (Dankel et al., 2010). Additionally, *EMX2* was identified as differentially methylated between human subcutaneous tissue and visceral adipose tissue (Keller et al., 2017).

The male DcoExp orangered1 module harboured one gene from the list of selected genes in the discriminant analysis: *LOC121818175* (small integral membrane protein 13-like). *SMIM13* was previously reported to be mapped within selection signature regions in Chinese Wagyu cattle (Wang et al., 2019). Several enriched GO terms associated with the response to insulin and regulation of fatty acids were obtained for the list of genes allocated within this module.

In total, five genes were allocated within the three female DcoExp modules. Only one gene was assigned to the palevioletred2 module, *LOC10560597* (tumour necrosis factor receptor superfamily member 26-like) and no direct association between *LOC10560597* and fat metabolism was found. Collagen type VI alpha 6 chain (*COL6A6*) and *BUB1* were present on the cyan module. In the lightcoral module, the genes *LOC101113771* (C-C motif chemokine 3-like) and *LOC114113921* (KATNB1-like protein 1) were present.

The *BUB1* gene was previously discussed, as this gene was also allocated to the lightslateblue male module. In humans, the *COL6A6* gene was identified as differentially expressed between insulin-resistant *versus* insulin-sensitive obese humans, obese individuals with macrophage crown-like structures *versus* obese individuals without macrophage crown-like structures, and obese *versus* nonobese individuals (Hardy et al., 2011; Lê et al., 2011; García-Alonso et al., 2016).

For the lightcoral female module, *LOC101113771* is predicted to be a chemokine-like gene, specifically, C-C motif chemokine 3 (*CCL3*). Chemokines are associated with the function of adipocytes as immune regulatory cells, showing an interesting expression pattern in adipose tissue (Bruun et al., 2005; Dahlman et al., 2005; Tilg and Moschen, 2006; Maury et al., 2007; Vielma et al., 2013). In humans, the expression of *CCL3* was higher in subcutaneous and visceral fat of obese patients, and its expression in subcutaneous tissue was positively correlated with fasting insulin concentration in serum (Huber et al., 2008).

In summary, 22 genes were simultaneously presented in the DcoExp modules significantly correlated with the fat percentage in different deposits and in the list of 314 genes selected in the DIABLO analysis. Among these 22 genes, eight were identified as potential functional candidate genes for fat metabolism-related processes through literature review (*RASSF5*, *COL1A1*, *PLA2G5*, *BUB1*, *EMEX2*, *LOC121818175*, *COL6A6* and *LOC10111377*). It is important to mention that the results obtained here may be interpreted carefully due to the sample size, which might affect the detection power. However, using different omics technologies with similar results and the criteria applied to the selection of candidate genes has the potential to reduce the sample size impact. Consequently, the methodology employed in the current study provides a link between genes that efficiently discriminate males and females (based on the expression and methylation pattern) with biological functions and production traits associated with meat and carcass quality in sheep.

## Conclusion

In the current study, the discriminant analysis performed using methylation patterns and expression values of genes harboring DMRs allowed perfect discrimination between male and female samples. The functional investigation of these genes in the context of co-expressed gene modules suggested an association with relevant biological processes involved in regulating production and meat quality traits. Indeed, these modules were associated with percentages of fat in different body deposits, reinforcing the potential functionality of these modules and the relevance of these genes. These results corroborate the initial hypothesis that an experimental design contrasting sexes might help identify candidate genes responsible for controlling meat quality traits of sheep products due to the importance of adipose deposits and the differences observed between males and females. Consequently, the results presented here pinpointed interesting functional candidate genes for fat percentage in different fat deposits in sheep.

## Data Availability

The data presented in the study are deposited in the Array Express (RNA-Seq: https://www.ebi.ac.uk/biostudies/arrayexpress/studies/E-MTAB-12130) and European Nucleotide Archive (WGBS: WGBS data: https://www.ebi.ac.uk/ena/browser/view/PRJEB55533) repositories, under the accession numbers E-MATB-12130 and PRJEB55533, respectively.
