# Supplementary material for: Integrated analyses of the methylome and transcriptome to unravel sex differences in the perirenal fat from suckling lambs
Source: Front Genet. 2022 Nov 1;13:1035063. doi: 10.3389/fgene.2022.1035063 (PMC9663842; doi:10.3389/fgene.2022.1035063)

Skyblue4 Male

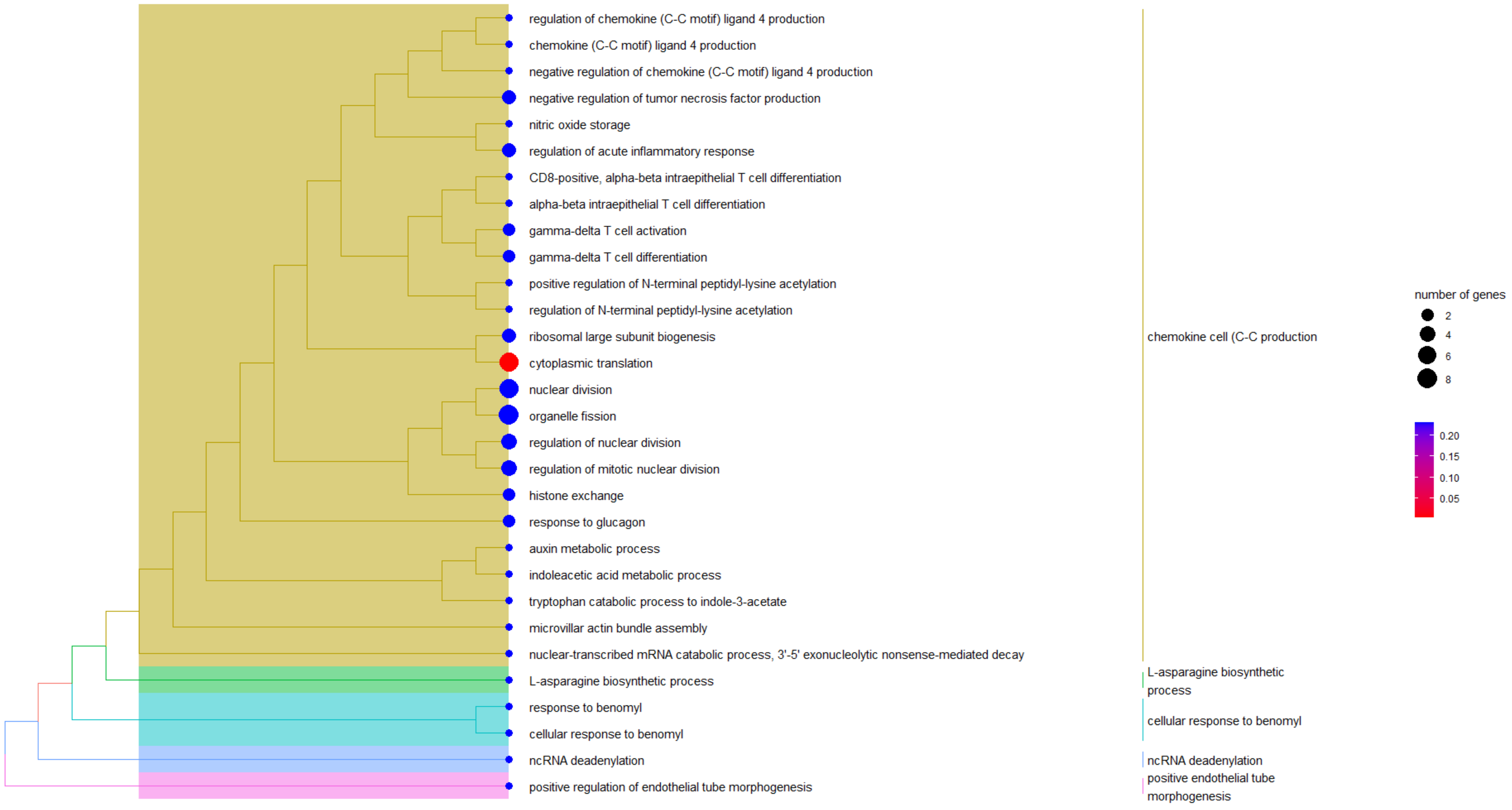

Lightslateblue Male

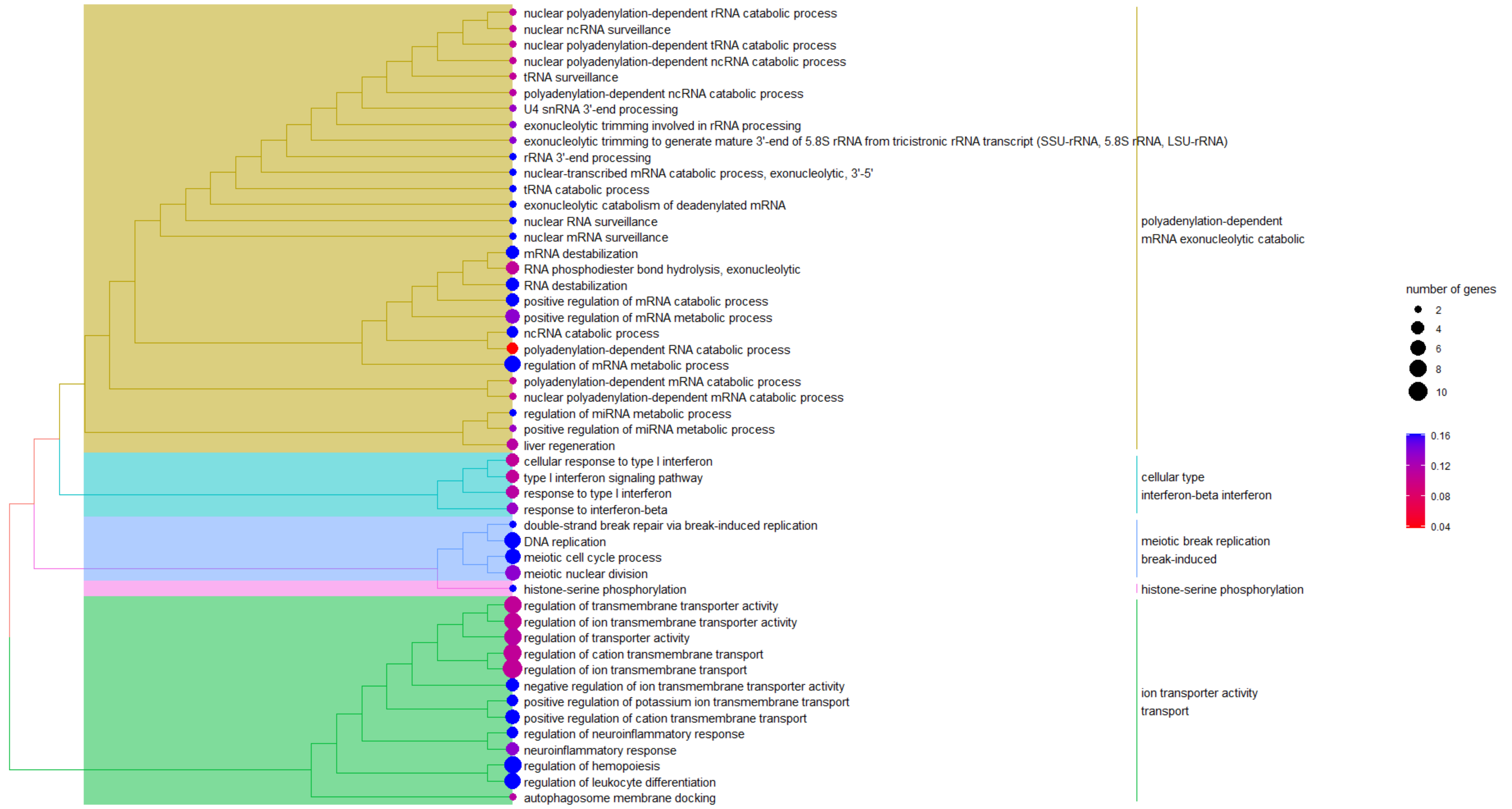

Magenta Male

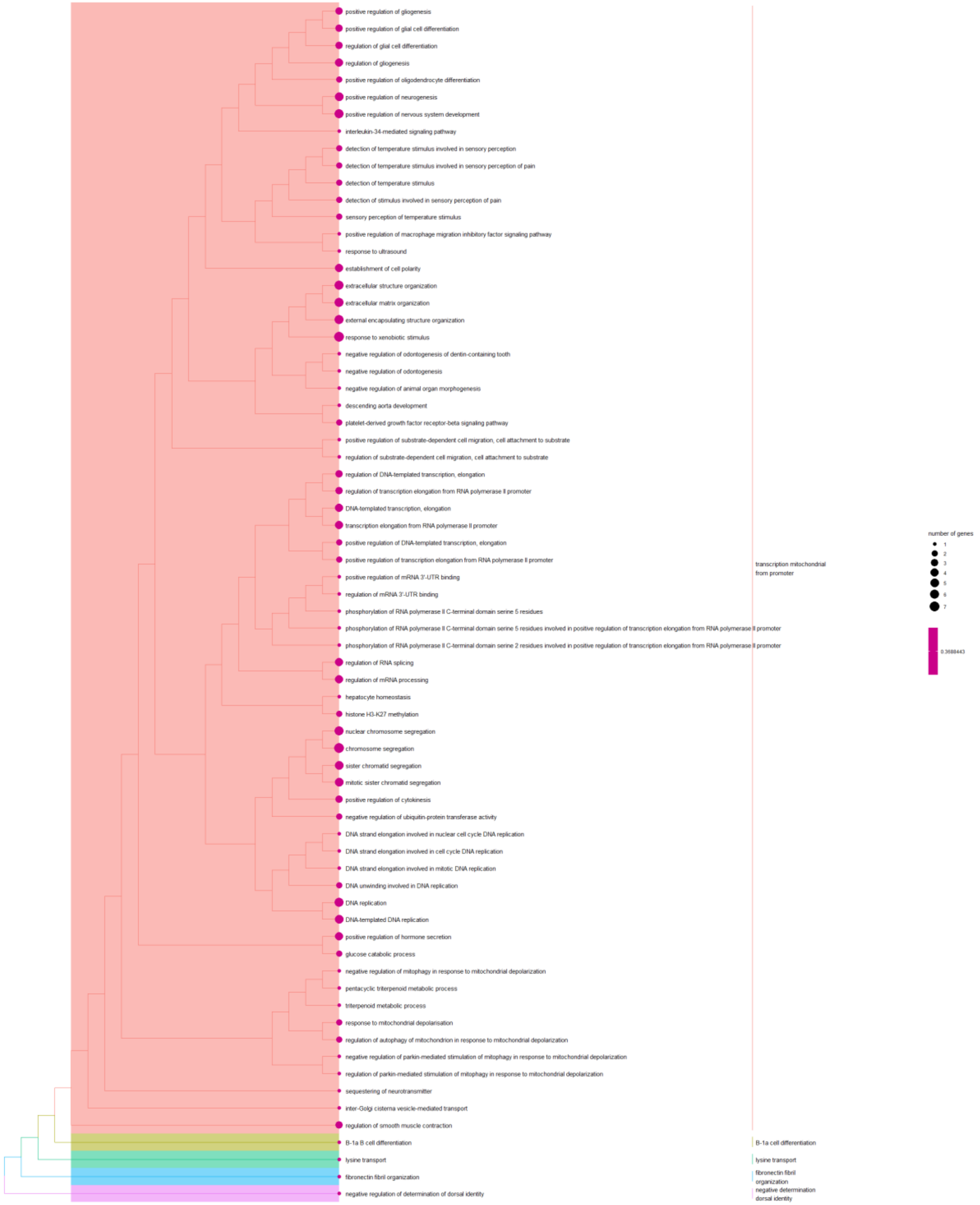

Darkorange Male

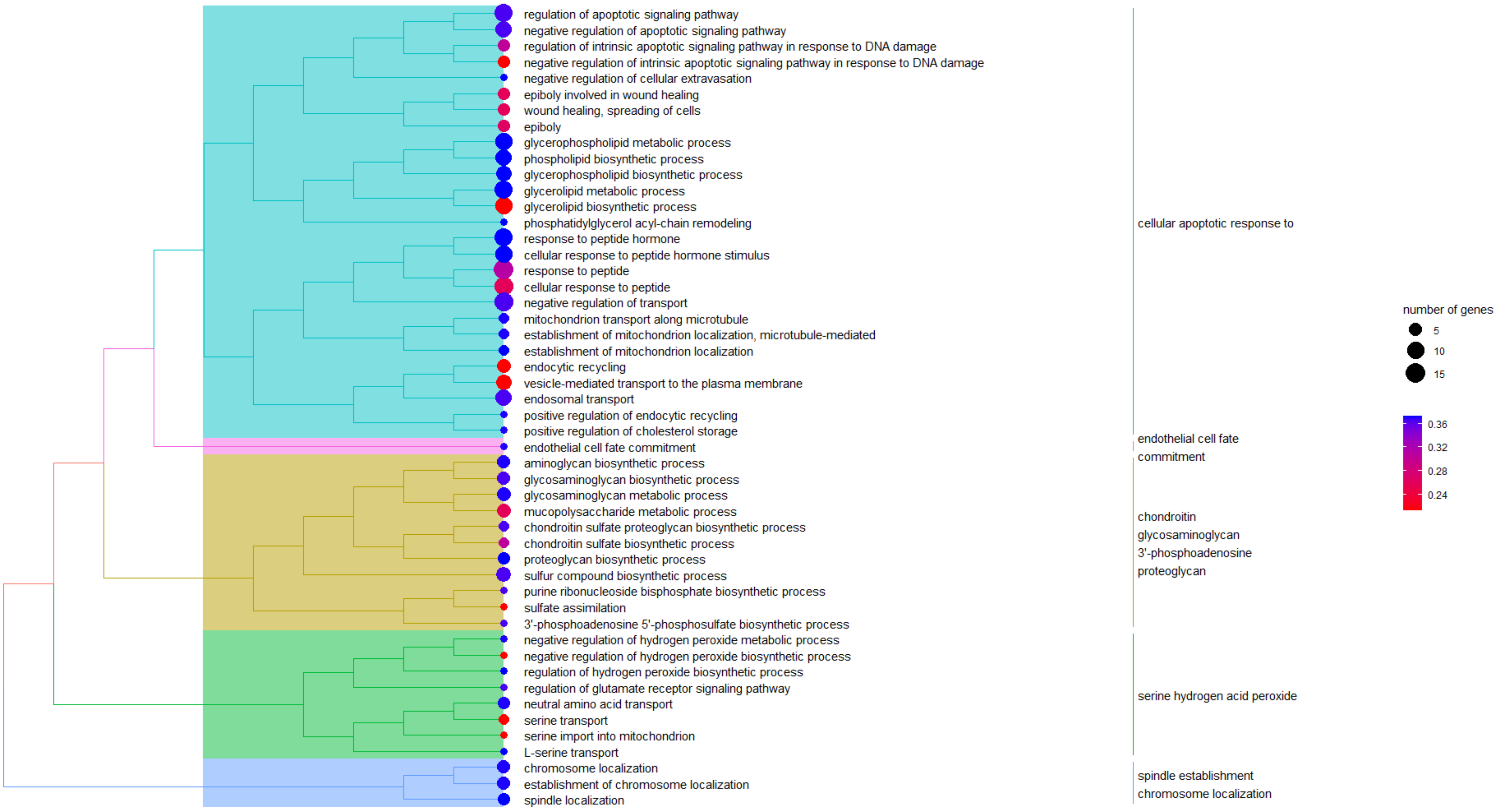

Brown4 Male

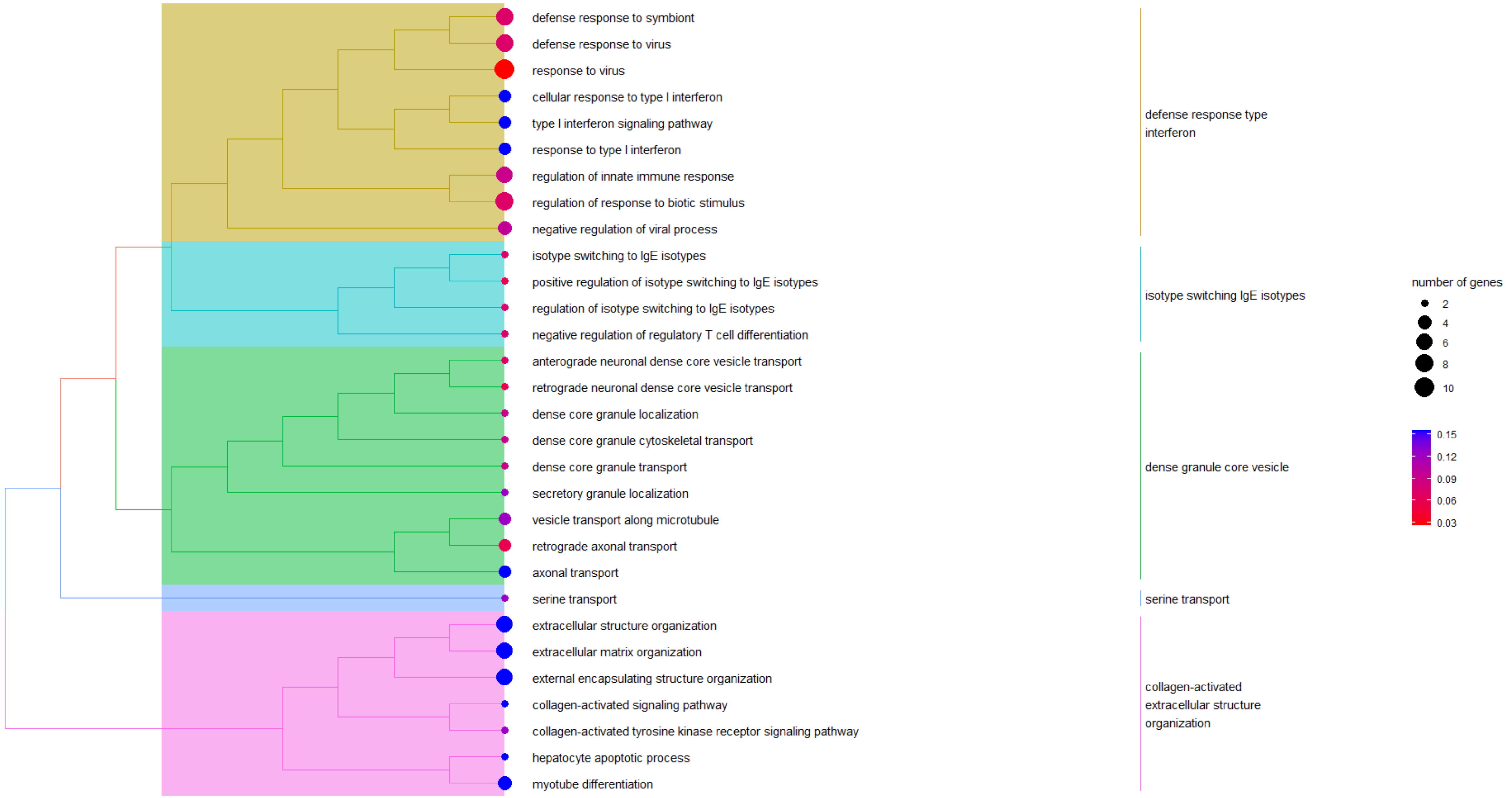

defense response to symbiont
defense response to virus
response to virus
cellular response to type I interferon
type I interferon signaling pathway
response to type I interferon
regulation of innate immune response
regulation of response to biotic stimulus
negative regulation of viral process
isotype switching to IgE isotypes
positive regulation of isotype switching to IgE isotypes
regulation of isotype switching to IgE isotypes
negative regulation of regulatory T cell differentiation
anterograde neuronal dense core vesicle transport
retrograde neuronal dense core vesicle transport
dense core granule localization
dense core granule cytoskeletal transport
dense core granule transport
secretory granule localization
vesicle transport along microtubule
retrograde axonal transport
axonal transport
serine transport
extracellular structure organization
extracellular matrix organization
external encapsulating structure organization
collagen-activated signaling pathway
collagen-activated tyrosine kinase receptor signaling pathway
hepatocyte apoptotic process
myotube differentiation
defense response type interferon
isotype switching IgE isotypes
dense granule core vesicle
serine transport
collagen-activated extracellular structure organization
number of genes
2
4
6
8
10
0.15
0.12
0.09
0.06
0.03

Orangered1 Male

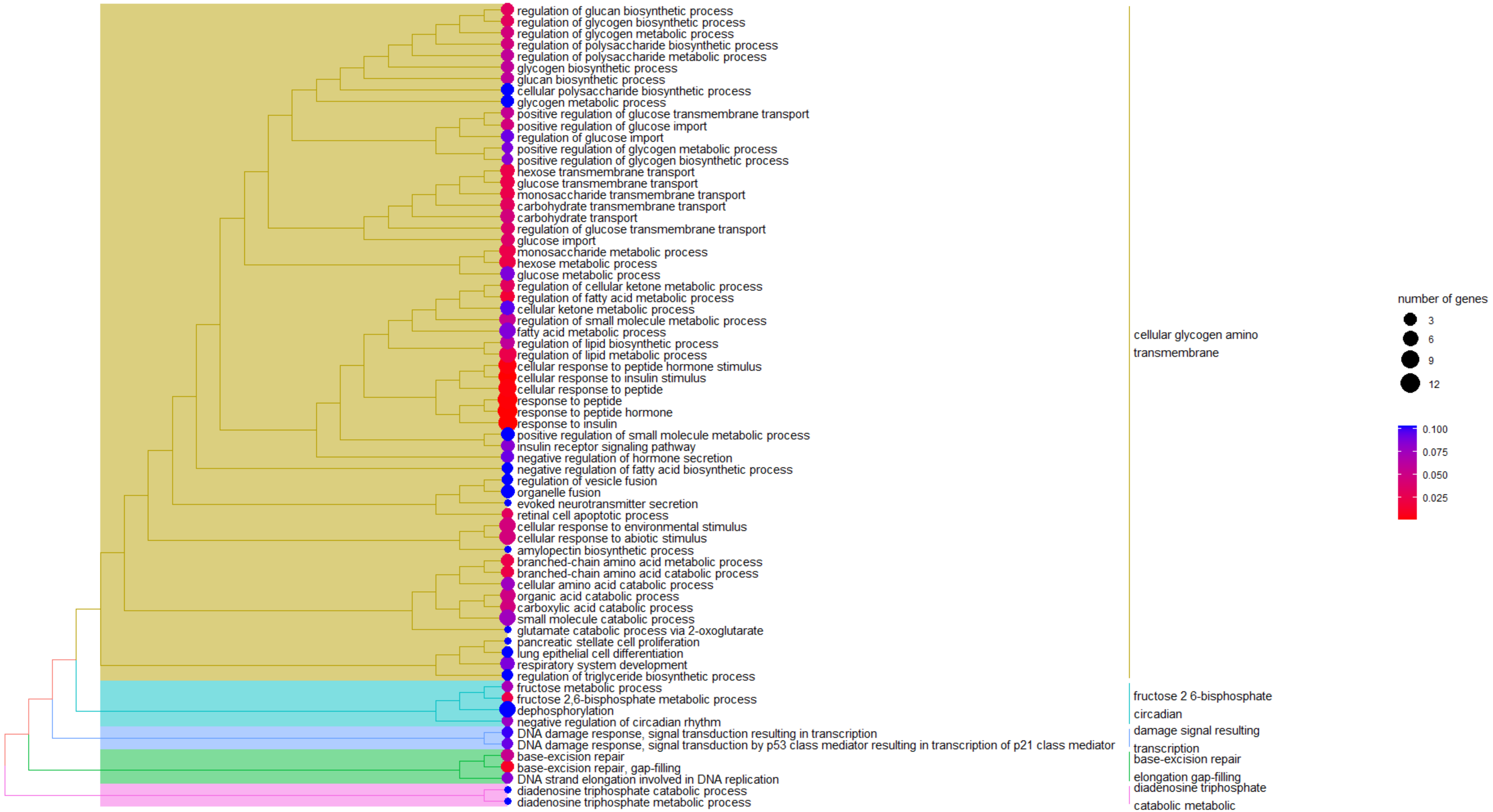

Lightblue4 Male

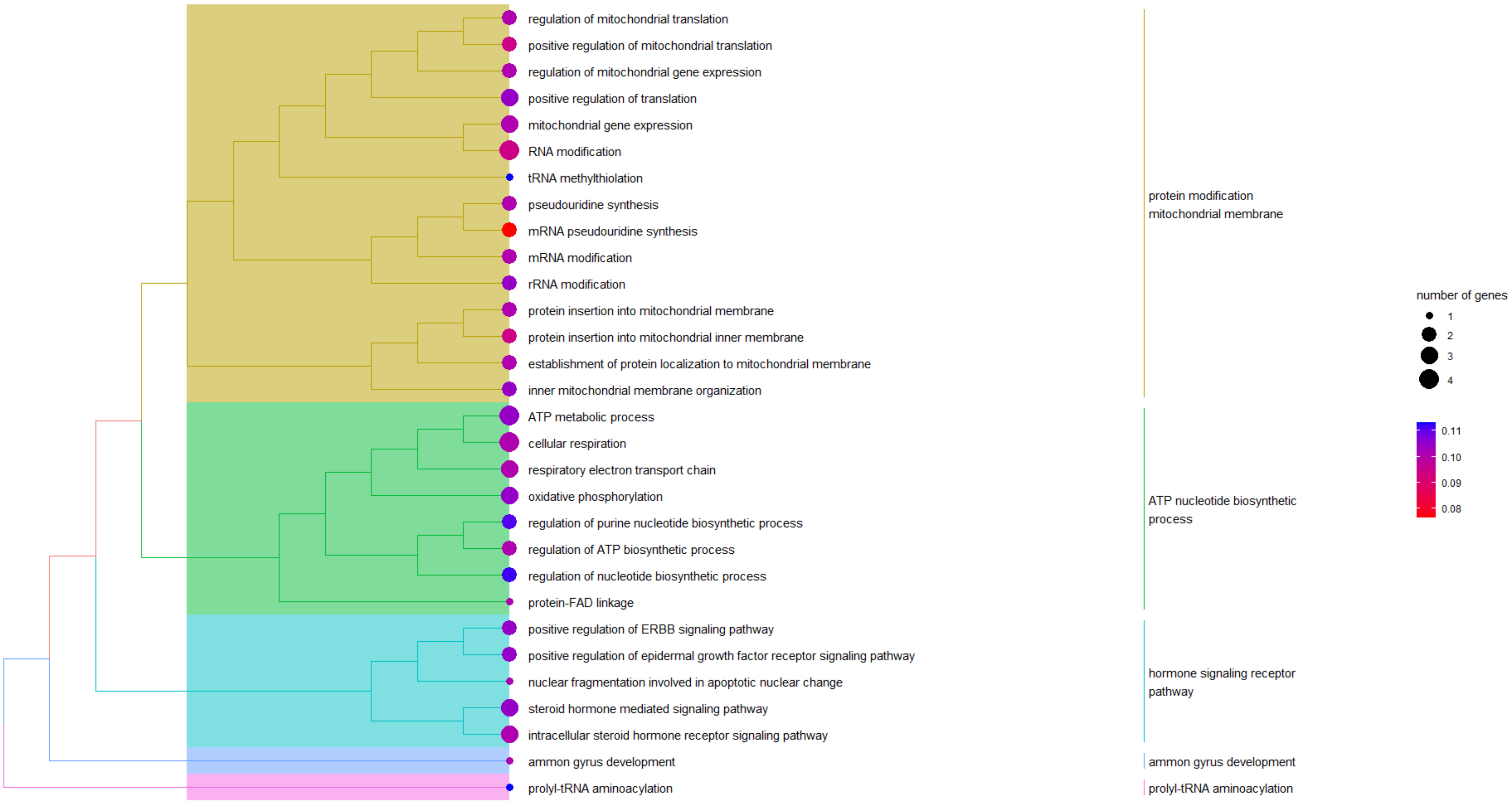

Lightpink4 Male

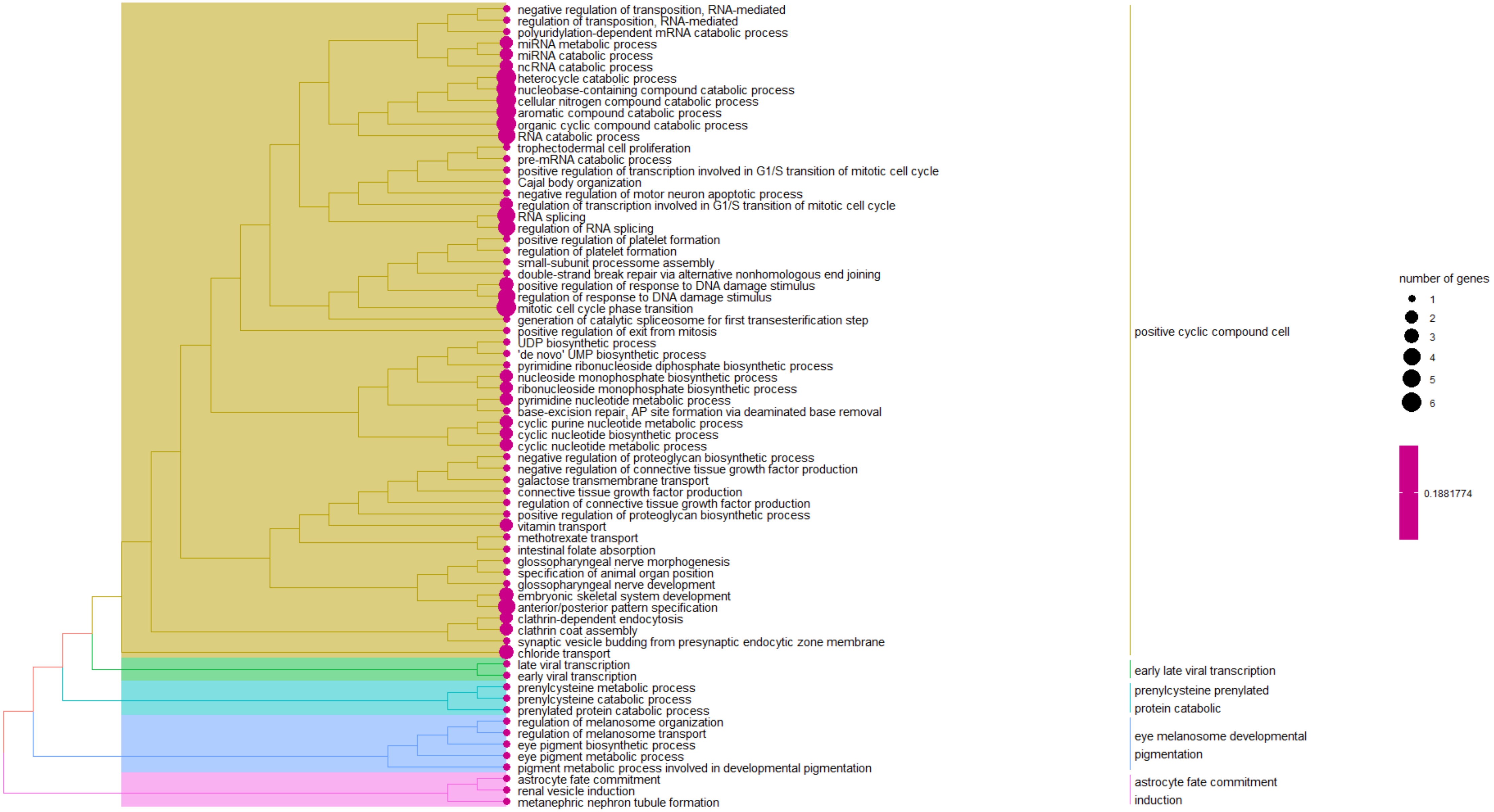

Coral1 Male

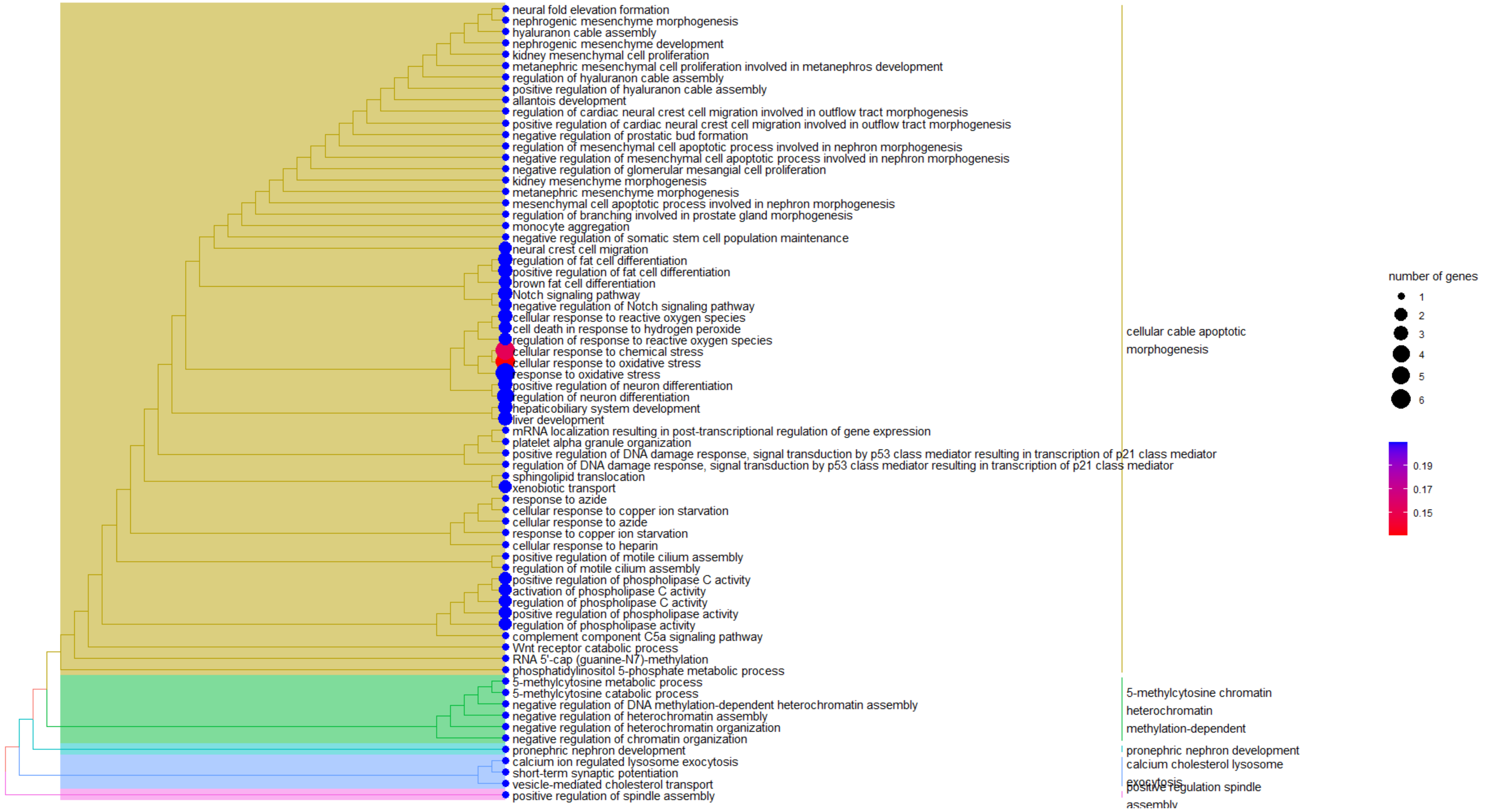

neural fold elevation formation
nephrogenic mesenchyme morphogenesis
hyaluranon cable assembly
nephrogenic mesenchyme development
kidney mesenchymal cell proliferation
metanephric mesenchymal cell proliferation involved in metanephros development
regulation of hyaluranon cable assembly
positive regulation of hyaluranon cable assembly
allantois development
regulation of cardiac neural crest cell migration involved in outflow tract morphogenesis
positive regulation of cardiac neural crest cell migration involved in outflow tract morphogenesis
negative regulation of prostatic bud formation
regulation of mesenchymal cell apoptotic process involved in nephron morphogenesis
negative regulation of mesenchymal cell apoptotic process involved in nephron morphogenesis
negative regulation of glomerular mesangial cell proliferation
kidney mesenchyme morphogenesis
metanephric mesenchyme morphogenesis
mesenchymal cell apoptotic process involved in nephron morphogenesis
regulation of branching involved in prostate gland morphogenesis
monocyte aggregation
negative regulation of somatic stem cell population maintenance
neural crest cell migration
regulation of fat cell differentiation
positive regulation of fat cell differentiation
brown fat cell differentiation
Notch signaling pathway
negative regulation of Notch signaling pathway
cellular response to reactive oxygen species
cell death in response to hydrogen peroxide
regulation of response to reactive oxygen species
cellular response to chemical stress
cellular response to oxidative stress
response to oxidative stress
positive regulation of neuron differentiation
regulation of neuron differentiation
hepaticobiliary system development
liver development
mRNA localization resulting in post-transcriptional regulation of gene expression
platelet alpha granule organization
positive regulation of DNA damage response, signal transduction by p53 class mediator resulting in transcription of p21 class mediator
regulation of DNA damage response, signal transduction by p53 class mediator resulting in transcription of p21 class mediator
sphingolipid translocation
xenobiotic transport
response to azide
cellular response to copper ion starvation
cellular response to azide
response to copper ion starvation
cellular response to heparin
positive regulation of motile cilium assembly
regulation of motile cilium assembly
positive regulation of phospholipase C activity
activation of phospholipase C activity
regulation of phospholipase C activity
positive regulation of phospholipase activity
regulation of phospholipase activity
complement component C5a signaling pathway
Wnt receptor catabolic process
RNA 5'-cap (guanine-N7)-methylation
phosphatidylinositol 5-phosphate metabolic process
5-methylcytosine metabolic process
5-methylcytosine catabolic process
negative regulation of DNA methylation-dependent heterochromatin assembly
negative regulation of heterochromatin assembly
negative regulation of heterochromatin organization
negative regulation of chromatin organization
pronephric nephron development
calcium ion regulated lysosome exocytosis
short-term synaptic potentiation
vesicle-mediated cholesterol transport
positive regulation of spindle assembly
cellular cable apoptotic morphogenesis
5-methylcytosine chromatin heterochromatin methylation-dependent
pronephric nephron development
calcium cholesterol lysosome exocytosis
positive regulation spindle assembly
number of genes
1
2
3
4
5
6
0.19
0.17
0.15

Cyan Female

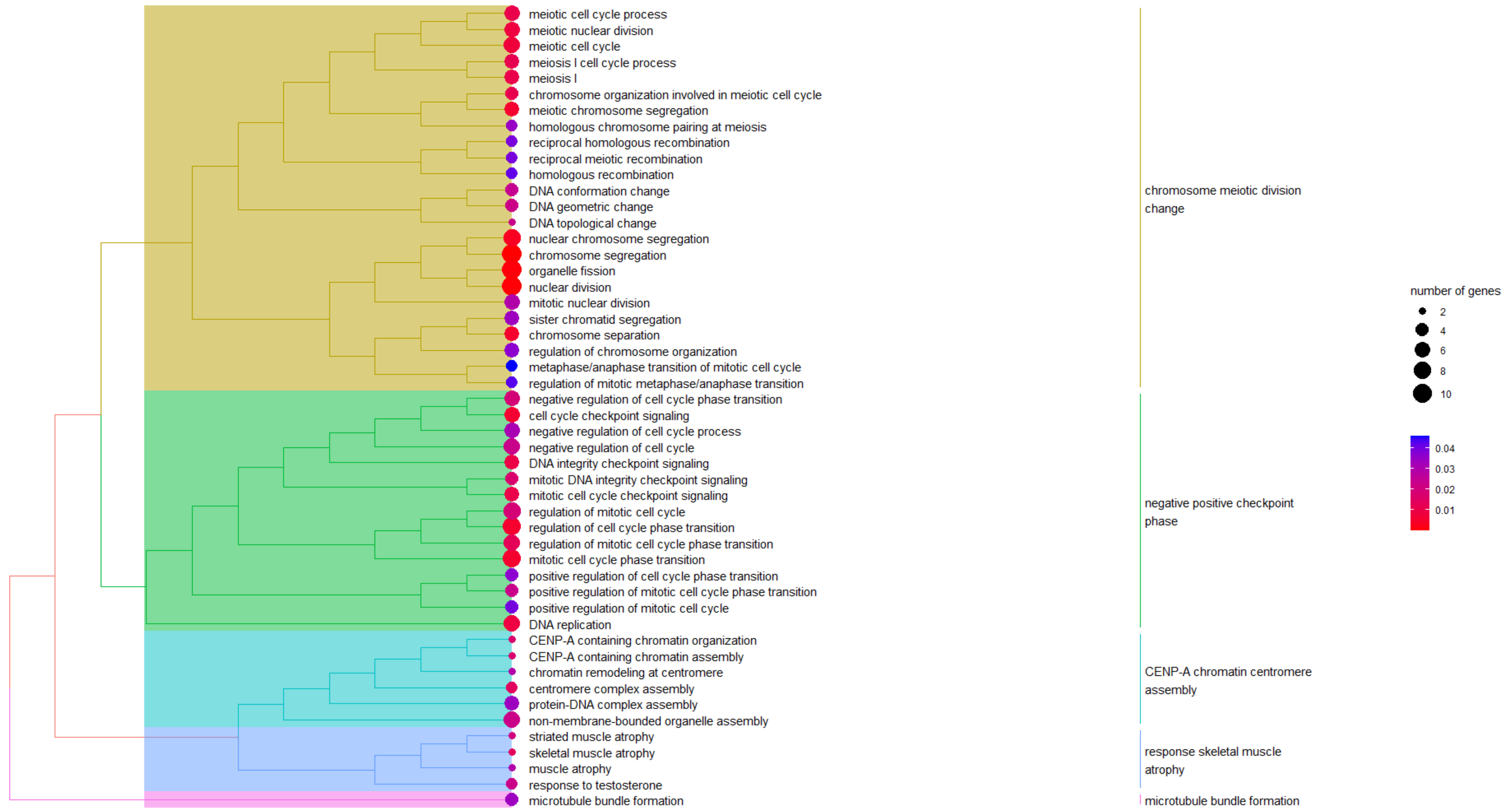

meiotic cell cycle process
meiotic nuclear division
meiotic cell cycle
meiosis I cell cycle process
meiosis I
chromosome organization involved in meiotic cell cycle
meiotic chromosome segregation
homologous chromosome pairing at meiosis
reciprocal homologous recombination
reciprocal meiotic recombination
homologous recombination
DNA conformation change
DNA geometric change
DNA topological change
nuclear chromosome segregation
chromosome segregation
organelle fission
nuclear division
mitotic nuclear division
sister chromatid segregation
chromosome separation
regulation of chromosome organization
metaphase/anaphase transition of mitotic cell cycle
regulation of mitotic metaphase/anaphase transition
negative regulation of cell cycle phase transition
cell cycle checkpoint signaling
negative regulation of cell cycle process
negative regulation of cell cycle
DNA integrity checkpoint signaling
mitotic DNA integrity checkpoint signaling
mitotic cell cycle checkpoint signaling
regulation of mitotic cell cycle
regulation of cell cycle phase transition
regulation of mitotic cell cycle phase transition
mitotic cell cycle phase transition
positive regulation of cell cycle phase transition
positive regulation of mitotic cell cycle phase transition
positive regulation of mitotic cell cycle
DNA replication
CENP-A containing chromatin organization
CENP-A containing chromatin assembly
chromatin remodeling at centromere
centromere complex assembly
protein-DNA complex assembly
non-membrane-bounded organelle assembly
striated muscle atrophy
skeletal muscle atrophy
muscle atrophy
response to testosterone
microtubule bundle formation
chromosome meiotic division change
negative positive checkpoint phase
CENP-A chromatin centromere assembly
response skeletal muscle atrophy
microtubule bundle formation
number of genes
2
4
6
8
10
0.04
0.03
0.02
0.01

Lightcoral Female

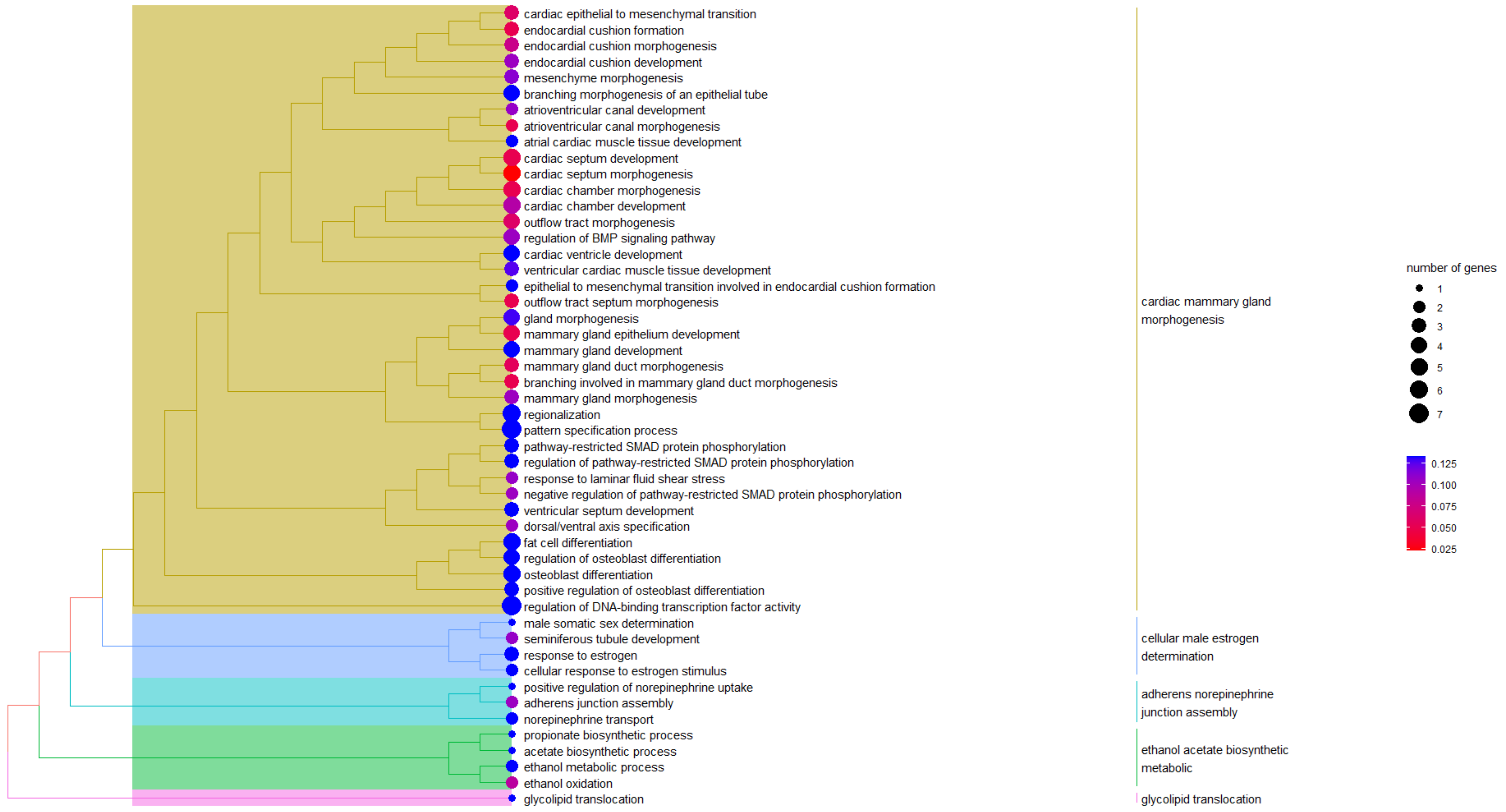

Palevioletred2 Female

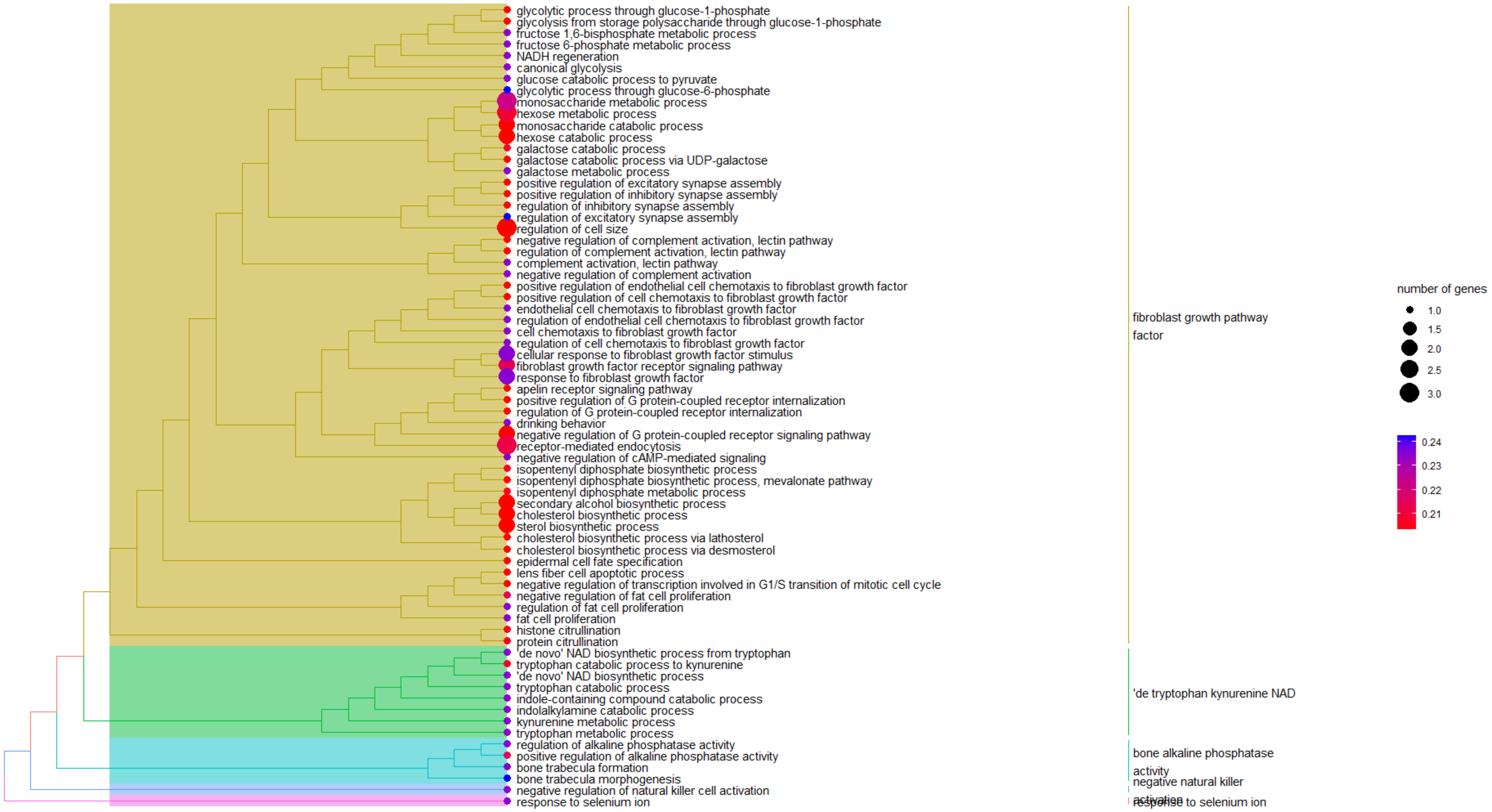

Supplement: Supplementary file 1 [file DataSheet1.ZIP › SupplementaryFigure2.pdf]
