# Supplementary material for: Integrated analyses of the methylome and transcriptome to unravel sex differences in the perirenal fat from suckling lambs
Source: Front Genet. 2022 Nov 1;13:1035063. doi: 10.3389/fgene.2022.1035063 (PMC9663842; doi:10.3389/fgene.2022.1035063)
Supplement: Supplementary file 1 [file DataSheet1.ZIP › SupplementaryTable1.docx]

Supplementary Table 1: Descriptive statistic of fat percentage in different fat deposits (Renal, Pelvic, Renal and Pelvic, Leg subcutaneous, and Leg intramuscular) measured in the half carcass for male and female Assaf suckling lambs.

| Trait | Mean (±SD) | | p-value |
| --- | --- | --- | --- |
|  | Male | Female |  |
| HCW (grams) | 2840.000 (±368.565) | 2696.667 (±80.416) | 0.391 |
| Renal (% on HCW) | 1.420  (±0.350) | 1.410 (±0.360) | 0.96 |
| Pelvic (% on HCW) | 2.835  (±0.727) | 1.999 (±0.487) | 0.045 |
| Renal and pelvic (% on HCW) | 2.374  (±0.609) | 2.061 (±0.409) | 0.323 |
| Leg intermuscular (% on HCW) | 3.698  (±0.688) | 3.353 (±0.846) | 0.457 |
| Leg subcutaneous (% on HCW) | 6.202  (±1.907) | 4.694 (±1.076) | 0.130 |
| Lumbar region subcutaneous (% on HCW) | 0.673  (±0.166) | 0.555 (±0.122) | 0.194 |

HCW: Half carcass weight; SD: Standard deviation; p-value: t-student p-value
