# Supplementary material for: Integrated analyses of the methylome and transcriptome to unravel sex differences in the perirenal fat from suckling lambs
Source: Front Genet. 2022 Nov 1;13:1035063. doi: 10.3389/fgene.2022.1035063 (PMC9663842; doi:10.3389/fgene.2022.1035063)
Supplement: Supplementary file 1 [file DataSheet1.ZIP › SupplementaryTable2.docx]

Supplementary Table 2: Mapping statistics for the RNA-sequencing data from perirenal fat of male and female Assaf suckling lambs.

| Sample | Percentage of uniquely mapped reads | Total number of reads |
| --- | --- | --- |
| Female 1 | 93.49 | 24527049 |
| Female 2 | 93.29 | 24294603 |
| Female 3 | 93.69 | 25868040 |
| Female 4 | 92.93 | 23490714 |
| Female 5 | 92.72 | 24145721 |
| Female 6 | 93.08 | 24006707 |
| Male 1 | 93.6 | 23672094 |
| Male 2 | 92.78 | 22195737 |
| Male 3 | 92.73 | 23738092 |
| Male 4 | 93.58 | 23318686 |
| Male 5 | 92.55 | 23140181 |
| Male 6 | 92.78 | 21948781 |
