# Supplementary material for: Integrated analyses of the methylome and transcriptome to unravel sex differences in the perirenal fat from suckling lambs
Source: Front Genet. 2022 Nov 1;13:1035063. doi: 10.3389/fgene.2022.1035063 (PMC9663842; doi:10.3389/fgene.2022.1035063)
Supplement: Supplementary file 1 [file DataSheet1.ZIP › SupplementaryTable5.docx]

Supplementary Table 5: Mapping statistics for the Whole Genome Bisulfite Sequencing data from perirenal fat of male and female Assaf suckling lambs.

| Sex | Mapping | mapped | no_mapped | mCG | mCHG | mCHH |
| --- | --- | --- | --- | --- | --- | --- |
| Female 1 | 0.9985 | 45261943937 | 65113391 | 72.049 | 1.391 | 1.439 |
| Female 2 | 0.9986 | 40792611972 | 55824328 | 71.441 | 1.410 | 1.462 |
| Female 3 | 0.9985 | 38653334171 | 55684886 | 71.341 | 1.400 | 1.471 |
| Female 4 | 0.9984 | 44650037081 | 68680792 | 70.128 | 1.414 | 1.470 |
| Female 5 | 0.9985 | 40052139899 | 56162707 | 70.929 | 1.401 | 1.500 |
| Female 6 | 0.9985 | 38848013809 | 55622815 | 71.552 | 1.377 | 1.449 |
| Male 1 | 0.9986 | 38925952857 | 53340477 | 71.149 | 1.426 | 1.497 |
| Male 2 | 0.9985 | 38813918939 | 55086893 | 71.703 | 1.410 | 1.470 |
| Male 3 | 0.9987 | 40289211283 | 50887893 | 71.762 | 1.467 | 1.523 |
| Male 4 | 0.9985 | 38149694417 | 56951086 | 70.823 | 1.399 | 1.462 |
| Male 5 | 0.9985 | 40530633198 | 60203108 | 71.884 | 1.390 | 1.446 |
| Male 6 | 0.9984 | 42877036781 | 65731404 | 71.224 | 1.366 | 1.415 |
