# Supplementary figures and images for: Integrated analyses of the methylome and transcriptome to unravel sex differences in the perirenal fat from suckling lambs
Source: Front Genet. 2022 Nov 1;13:1035063. doi: 10.3389/fgene.2022.1035063 (PMC9663842; doi:10.3389/fgene.2022.1035063)

### Correspondence of Male vs Female set-specific modules

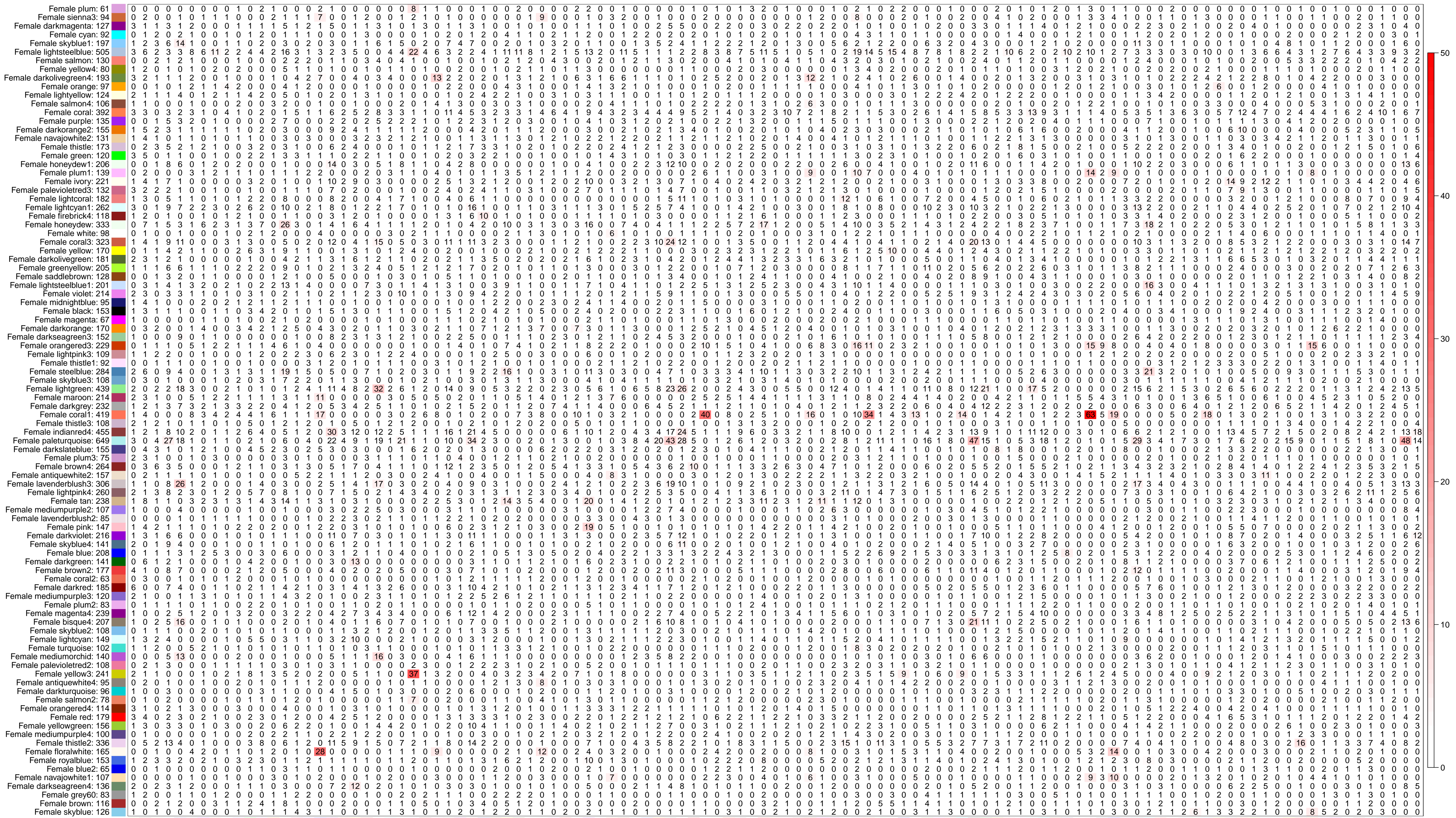

Supplement: Supplementary file 1 [file DataSheet1.ZIP › SupplementaryFigure1.pdf]
